# Ligand-guided homology modelling of the GABA_B2_ subunit of the GABA_B_ receptor

**DOI:** 10.1371/journal.pone.0173889

**Published:** 2017-03-21

**Authors:** Thibaud Freyd, Dawid Warszycki, Stefan Mordalski, Andrzej J. Bojarski, Ingebrigt Sylte, Mari Gabrielsen

**Affiliations:** 1 Department of Medical Biology, Faculty of Health Sciences, UiT - the Arctic University of Norway, Tromsø, Norway; 2 Department of Medicinal Chemistry, Institute of Pharmacology, Polish Academy of Sciences, Kraków, Poland; University of Parma, ITALY

## Abstract

γ-aminobutyric acid (GABA) is the main inhibitory neurotransmitter in the central nervous system, and disturbances in the GABAergic system have been implicated in numerous neurological and neuropsychiatric diseases. The GABA_B_ receptor is a heterodimeric class C G protein-coupled receptor (GPCR) consisting of GABA_B1a/b_ and GABA_B2_ subunits. Two GABA_B_ receptor ligand binding sites have been described, namely the orthosteric GABA binding site located in the extracellular GABA_B1_ Venus fly trap domain and the allosteric binding site found in the GABA_B2_ transmembrane domain. To date, the only experimentally solved three-dimensional structures of the GABA_B_ receptor are of the Venus fly trap domain. GABA_B_ receptor allosteric modulators, however, show great therapeutic potential, and elucidating the structure of the GABA_B2_ transmembrane domain may lead to development of novel drugs and increased understanding of the allosteric mechanism of action. Despite the lack of x-ray crystal structures of the GABA_B2_ transmembrane domain, multiple crystal structures belonging to other classes of GPCRs than class A have been released within the last years. More closely related template structures are now available for homology modelling of the GABA_B_ receptor. Here, multiple homology models of the GABA_B2_ subunit of the GABA_B_ receptor have been constructed using templates from class A, B and C GPCRs, and docking of five clusters of positive allosteric modulators and decoys has been undertaken to select models that enrich the active compounds. Using this ligand-guided approach, eight GABA_B2_ homology models have been chosen as possible structural representatives of the transmembrane domain of the GABA_B2_ subunit. To the best of our knowledge, the present study is the first to describe homology modelling of the transmembrane domain of the GABA_B2_ subunit and the docking of positive allosteric modulators in the receptor.

## Introduction

G-protein coupled receptors (GPCRs) belong to a superfamily of integral membrane proteins that are activated by a formidable variety of ligands–from photons and ions to neurotransmitters, lipids and peptides—and hence are involved in the regulation of a wide variety of cellular and physiological functions. Members of the GPCR superfamily are characterised by a canonical seven α-helical transmembrane (7TM) domain topology and their activation of cytoplasmic guanine nucleotide-binding proteins (G-proteins) upon receptor activation. However, there is great variability in both amino acid sequence and functional coupling among the > 800 human GPCR sequences (i.e., approx. 2% of the human genome) that have been identified [1]. Using exhaustive phylogenetic analysis, the GPCRs have been classified into the Glutamate (22 receptors), Rhodopsin (~680 receptors– 284 non-olfactory, ~380 olfactory), Adhesion (33 receptors), Frizzled (11 receptors), and Secretin (15 receptors), and Others (>40 receptors) families (the GRAFS classification system) [2,3]. The Rhodopsin, Secretin, Glutamate and Frizzled families also correspond to the class A, B, C, and F GPCRs, respectively, in the A-F classification system [4]. Until very recently, the only available x-ray crystal structure templates for homology modelling of the 7TM domain of GPCRs belonged to class A GPCRs. Within the last years, however, the number of experimentally solved GPCR structures have increased significantly, and x-ray crystal structures of nearly 40 unique GPCRs, including class B, C and F GPCRs, have now been released [5,6].

The GABA_B_ receptor is a class C GPCR and one of three native receptors of γ-aminobutyric acid (GABA), the other two being the ionotropic GABA_A_ and GABA_C_ receptors. The GABA_B_ receptor is located in both pre- and postsynaptic inhibitory and excitatory synapses as well as in perisynaptic and extrasynaptic plasma membranes [7]. While activation of postsynaptic GABA_B_ receptors results in opening of G-protein-coupled inwardly-rectifying potassium (GIRK) channels, hyperpolarising potassium conductance and direct inhibition of postsynaptic calcium channels, activation of presynaptic GABA_B_ activation results in reduction of neurotransmitter release, primarily through the inhibition of calcium-dependent neurotransmitter release [7]. GABA is the main inhibitory neurotransmitter of the central nervous system (CNS), and GABAergic inhibitory interneurons play pivotal roles in the process of cortical inhibition by attenuating the activities of other cortical neurons, in particular, the excitatory pyramidal neurons, and by generating inhibitory post-synaptic potentials (IPSPs) that modulate cortical excitability and neural plasticity [8]. Disturbances in the GABAergic system have been implicated in numerous neurological and neuropsychiatric disorders, including anxiety and depression, epilepsy, autism spectrum disorders, drug addiction, and schizophrenia, as well as other conditions such as muscle spasticity, gastrointestinal reflux disorder, and pain [9,10].

**Fig 1:**
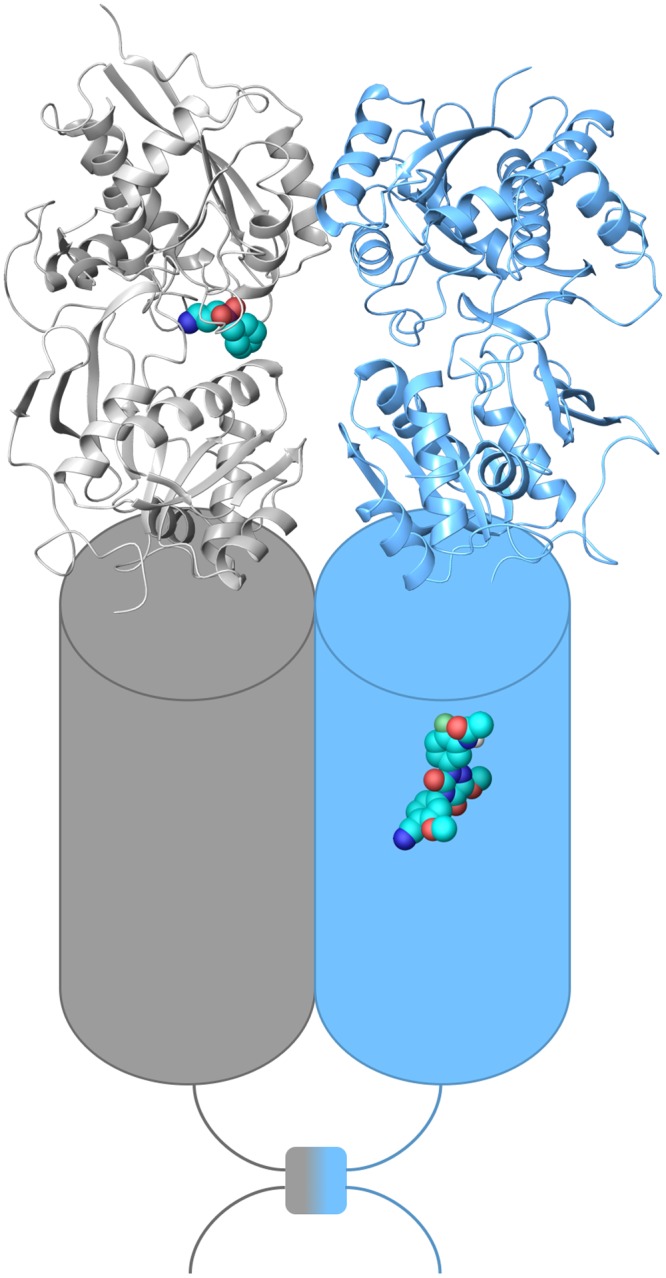
Schematic representation of the GABA_B1_ (grey) and GABA_B2_ (blue) subunits of the GABA_B_ receptor. X-ray crystal structure of extracellular VFT domain in complex with the antagonist CGP46381 (PDB id 4MS1) (ribbon representation), 7TM domain (cylinder representation) with the PAM ADX71943 representing the allosteric binding pocket, and intracellular C-terminal domain with the coil-coil interaction, are shown.

The GABA_B_ receptor is a functional heterodimer consisting of GABA_B1a/b_ and GABA_B2_ subunits [10]. A schematic illustration of the receptor is shown in Fig 1. Each subunit consists of three distinct domains, the N-terminal, extracellular domain, also known as the Venus fly trap (VFT) domain, the 7TM domain characteristic of all GPCRs, and the intracellular C-terminal tail [10]. The cysteine-rich domain (CRD) linking the VTF to the 7TM bundle in other class C members is absent in the GABA_B_ receptor [11]. Two GABA_B_ receptor ligand-binding sites have been characterised. The orthosteric binding site recognised by GABA and other agonists and antagonists is located within the extracellular VTF domain of the GABA_B1_ subunit [10]. To date, the only GABA_B_ receptor x-ray crystal structures available are of the VTF domain [12]. Likewise, the only currently marketed GABA_B_ receptor drug is the orthosteric agonist baclofen, a muscle relaxant and antispastic agent; however, the therapeutic use of baclofen is limited due to its low CNS permeability, short duration of action, narrow therapeutic window, and rapid tolerance [10].

The GABA_B_ receptor allosteric binding site has been mapped to the 7TM domain of GABA_B2_ receptor subunit (Fig 1) [13,14]. Binding of ligands to allosteric sites of GPCRs may cause receptor conformational changes that positively or negatively impact the affinity (association and/or dissociation rates) or the efficacy (intracellular responses) of the orthosteric ligand [15]. As allosteric modulators acting through affinity or efficacy modulation exert their effects only upon binding of an orthosteric agonist, they hence provide a fine-tuning of the physiological signal rather than turning the signal on or off themselves. Allosteric binding sites are also often less conserved than the orthosteric binding sites of homologous GPCRs, and allosteric modulators thus may act more selectively and potentially cause fewer side effects than orthosteric ligands. Due to their non-competitive mode of action, lower dosages of the ligands may also be administered to obtain the desired pharmacological effect, further reducing the risks of adverse side effects. GABA_B_ receptor studies have shown that receptor desensitisation and down-regulation is less likely to occur with positive GABA_B_ allosteric modulators than with orthosteric agonists, and have also indicated that positive allosteric modulators may be devoid of the adverse effects of the agonist baclofen [16]. Currently, only a limited number of GABA_B_ receptor positive allosteric modulators (PAMs), which act by enhancing the potency and efficacy of GABA_B_ receptor orthosteric ligands, have been identified (S1 Table). The first GABA_B_ receptor PAMs discovered were the di-*tert*-butylbenzenes and pyrimidines CGP7930 and GS39783 and their analogues, reported in 2001 and 2003 [17,18]. More lately, the thiophenes COR627, COR628 and analogues, and the structurally unrelated ADX71943 and analogues, have been shown to be GABA_B_ receptor PAMs [19,20]. In 2016, the structurally novel compound SSD114 was also shown to be a GABA_B_ receptor PAM [21]. The PAM ADX71441, whose structure first recently was disclosed [22], has moreover been granted regulatory approval to start Phase I clinical trials, where it will be investigated for therapeutic use in Charcot-Marie-Tooth Type 1A disease (CMT1A), alcohol use disorder, and nicotine dependence [23]. In addition, three analogues of the di-*tert*-butylbenzene PAMs were recently also identified as negative allosteric modulators (NAMs) of the GABA_B_ receptor, acting by decreasing the efficacy of the orthosteric ligands [24,25].

To gain a deeper understanding of the molecular mechanism of action of allosteric modulation of the GABA_B_ receptor and to aid the discovery of novel allosteric modulators of the receptor, knowledge about the three-dimensional (3D) structure of the allosteric binding site of the receptor is of crucial importance. In the present study, extensive homology modelling of the 7TM domain of GABA_B2_ subunit has been performed using templates from GPCR classes A, B, and C, followed by docking of GABA_B_ receptor PAMs and decoys to identify models that enrich the active compounds. This ligand-guided homology modelling approach has resulted in the selection of a subset of eight different homology models representing the GABA_B2_ 7TM domain that enrich the GABA_B_ receptor PAMs. By analysing the docking results of the PAMs in these models using structural interaction fingerprints (SIFt) and site-directed mutagenesis data, a detailed description of the putative allosteric binding site of the GABA_B_ receptor is provided.

## Methods

**Fig 2:**
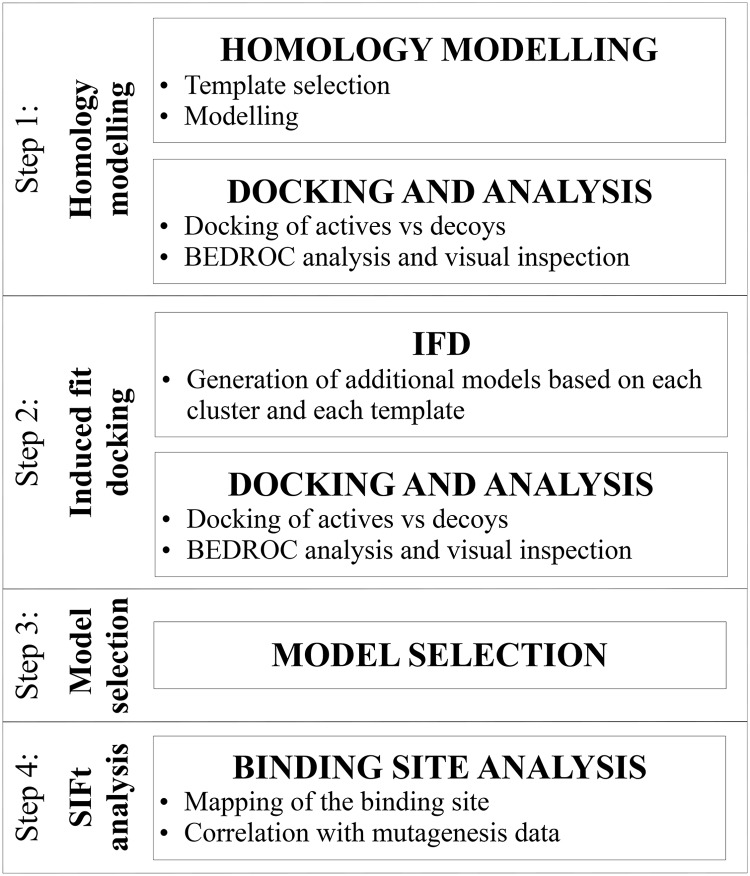
Overview of the ligand-guided homology modelling approach. IFD, induced-fit docking; BEDROC, Boltzmann-enhanced discrimination of receiver operating characteristic; SIFt, structural interaction fingerprints.

A schematic overview of the ligand-guided homology modelling approach used in the current paper can be found in Fig 2.

### Homology modelling

The following six x-ray crystals structures were used as templates for construction of homology models of the 7TM domain of the GABA_B2_ subunit: the class A bovine rhodopsin and β_2_-adrenergic receptors (β_2_-AR) (PDB ids 1U19 and 2RH1), the class B human corticotrophin release factor 1 (CRF1) and glucagon receptors (PDB ids 4K5Y and 4L6R), and the class C human metabotropic glutamate receptors 1 and 5 (mGlu1 and mGlu5, PDB ids 4OR2 and 4OO9). The rhodopsin and β_2_-AR x-ray crystal structures were included as templates for modelling of GABA_B2_ as they previously have been successfully used to model class C GPCRs [26–29]. In addition, two recently released class B x-ray crystal structures that shared approx. the same template-target sequence identity as the rhodopin-GABA_B2_ and β_2_-AR-GABA_B2_ were included as templates.

The Schrödinger Maestro multiple sequence viewer (MSV) tool [30] was used to construct a structure-based alignment of the six selected templates. The template structure TM domains were superimposed onto the TM domain of the mGlu1 x-ray crystal structure (PDB id 4OR2), and the sequences were aligned according to the structure superposition. Guided by the Wu class C and class A-class C alignments [31], manual adjustments of the template alignment was then performed to remove helical gaps generated by the structural alignment procedure and to correct other helical alignments errors. The template structures were then superimposed again according to the adjusted sequence alignment.

Following the alignment of the template structures, the amino acid sequences of the human GABA_B1_ and GABA_B2_ subunits (UniProt [32] accession numbers Q9UBS5 and O75899) were then added to the structure-based alignment, and for each of the six templates, a template-GABA_B2_ sequence alignment was extracted. The start and end of the 7TM helices were manually adjusted to fit the length of the template TMs, and the loops of the template and target sequences were unaligned, except the regions around the cysteine in extracellular loop 2 (EL2). The final template-GABA_B2_ 7TM alignments used for construction of the GABA_B2_ homology models are found in S1 Fig.

MODELLER software version 9.13 [33] was used to construct and refine the GABA_B2_ homology models. During model construction, TM6 of the class B-based models was specified to consist of residues in positions 6.35x35-6.57x57 (GPCR database (GPCRdb) numbering scheme, see below) to include residues G706^6.53x53^, A708^6.55x55^, and S710^6.57x57^, which have been shown to be important for PAM binding in the GABA_B_ receptor through site-directed mutagenesis [14]. Visual inspection of the initial GABA_B2_ models constructed based on class C templates also revealed that ELs 1 and 2 were entwined. Due to EL2 containing residue C648 involved in the conserved disulphide bond with C553^3.29x29^ (TM3), EL1 was not included in these models. In total, 600 GABA_B2_ homology models (100 models per template) were generated. To minimise violations of the spatial constraints, conjugate gradient energy minimization followed by MD annealing (iterative steps of heating (to 1300 K) and cooling (to 300 K)) was performed using the refine.very_slow function of Modeller [33].

### Ligand-guided model selection

#### Ligands

**Fig 3:**
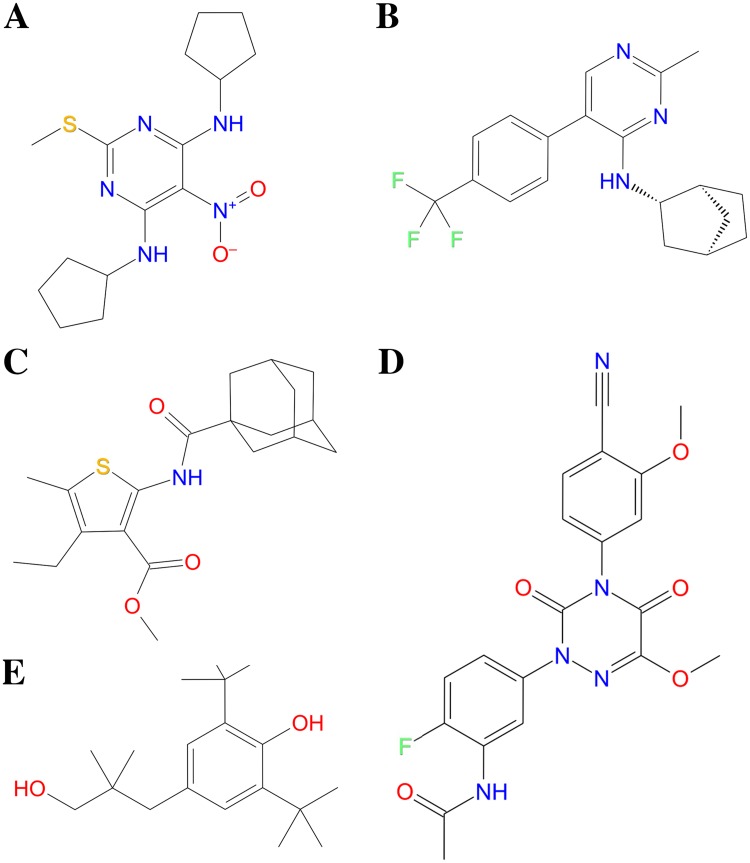
Structures of PAM cluster 1–5 representatives. (A) **GS39783** (Cluster 1), (B) **27** (**BHF177**) (Cluster 2), (C) **COR627** (Cluster 3), (D) **ADX71943** (Cluster 4), and (E) **rac-BHFF** (Cluster 5). Cluster 1 E_max_ range: 20%–78% (11 PAMs), cluster 2 EC_50_ range: 870–5000 nM (10 PAMs), cluster 3 range of increase (%) compared to 10μM [GABA]: 5.9%–19% (13 PAMs), cluster 4 EC_50_ range: <100–1000 nM (32 PAMs), and cluster 5 EC_50_ range: 4.6–4600 nM (6 PAMs). Please see S1 Table for full activity data.

72 unique PAMs were retrieved from the scientific literature and clustered using MOLDPRINT2D fingerprints, Tanimoto similarity metrics and average cluster linkage method using Schrödinger Canvas software [34]. Application of Kelly criterion [35] resulted in 17 clusters that were reduced to five distinct chemical clusters by merging the most similar ligand clusters. Full ligand activity data can be found in S1 Table. Structures of commonly studied PAMs from each cluster of actives are shown in Fig 3.

A combination of known inactive compounds and property-matched compounds were used as decoys in the ligand-guided model selection. 51 inactive analogues of the PAMs in clusters 2 and 3 were identified from the scientific literature (S2 Table) [36,37]. In addition, 2536 property-matched decoys (presumed inactive compounds with similar physicochemical properties but dissimilar 2D topology compared with the actives) were selected from the ZINC database [38] using an in-house script that followed the Directory of useful decoys (DUD) methodology [39]. Merging of the property-based and inactive compounds to one decoy set yielded PAM:decoy ratios ranging between approx. 1:83 (cluster 4) to approx. 1:431 (cluster 5).

#### Ligand docking

Prior to docking of the PAMs and decoys, the appropriate ionisation states at pH 7.4 were assigned to all ligands using Schrödinger Epik [40]. Ligand 3D structures were generated in Schrödinger Ligprep [41] using the OPLS2005 force field and generating one low energy ring conformation per ligand. For the PAMs and known inactive compounds, chiral centres specified in literature were kept while unspecified centres were labelled racemic.

The 600 GABA_B2_ homology models were prepared for docking using the one-step protein preparation workflow in Schrödinger Maestro [30] by adding and refining missing hydrogen atoms and adding the disulphide bridge between C553^3.29x29^ and C648^EL2^. GABA_B_ receptor site-directed mutagenesis data [14] was used to determine the GABA_B2_ allosteric site into which the ligands were docked. Receptor grid maps representing the shape and chemical properties of the binding site were generated using Schrödinger Glide [42] by centring on residue Y564^3.40x40^ as amino acids in position 3.40x40 in class C GPCRs correspond to position 3.36 in the class A GPCRs [5], a well-known protein-ligand interaction position. The corresponding residues in mGlu1 and mGlu5 crystal structures (S668 and P655, respectively) were also located in the allosteric binding site of these class C members [31,43,44]. Due to the differences in relative position between Y564^3.40x40^ and TM6 of the generated models, an outer grid box size of 25 Å (class A- and B-based models) or 30 Å (class C-based models) were used in the present study to ensure that the aforementioned residues G706^6.53x53^, A708^6.55x55^, and S710^6.57x57^ were included in the grid maps. An inner grid box size of 10 Å was used in all models.

Docking of the PAMs and decoys was performed using the Schrödinger Virtual Screening Workflow tool using Glide Standard Precision (SP) with the OPLS2005 force field [42]. One pose per ligand was kept for post-docking full force field minimisation (optimisation of ligand pose geometry followed by recalculation of interaction strength between ligand-protein using the scaled Coulomb-van der Waals term and the Glide score).

#### Docking analysis

To analyse the docking results, the Boltzmann-enhanced discrimination of receiver operating characteristic (BEDROC) method was employed [45]. BEDROC is a method that incorporates 'early recognition' (the α exponent prefactor) to weigh the contribution of rank to the final score [45]. To calculate the BEDROC score for each homology model based on the docking of the actives in each cluster versus the decoys, the compounds were ranked according to their Glide score and an α value of 20.0 was employed to ensure that 80% of the maximum contribution to the BEDROC score came from the first 8% of the ranked list, as recommended by Truchon et al. [45].

#### Induced Fit Docking (IFD)

To improve the docking results, the Schrödinger Induced Fit Docking (IFD) protocol [34] was applied. The input GABA_B2_-ligand complexes were selected by ranking the 100 models/template per cluster of actives according to their BEDROC scores and visually inspecting the docking results of the cluster of actives in each of the top-ranked models. The model that docked the highest numbers of PAMs, or where the binding mode of the active PAMs was the most consistent, was chosen. The ligand that best represented the observed binding mode of the PAMs in each chosen model, or the ligand with the largest volume, was then selected. As no common binding mode of the actives could be identified in the homology models based on the human glucagon receptor (PDB id 4L6R), GABA_B2_ models based on this template were not included. Hence, 25 GABA_B2_-ligand complexes (5 clusters of actives, 5 templates) were used as input for IFD (S3 Table).

For each of the final GABA_B2_-ligand complexes, the ligand was docked into its original model and maximum 50 different protein-ligand complexes were generated. Backbone and side-chain minimisation of the neighbouring residues within 5Å of the ligand in each new complex was then performed, followed by redocking of the ligand into maximum 20 new protein structures that were within 30 kcal/mol of the best refined structure. In total, the IFD protocol resulted in the generation of 456 new models of the GABA_B2_ subunit.

Two of the top-ranked pre-IFD mGlu5-based models docked all cluster 1 PAMs, but only in one of the models did the ligands dock completely and consistently into the allosteric site (results not shown). EL2 of this model was, however, incorrectly folded, and though the loop was distal to the allosteric ligand site and did not affect the docking orientations, the loop had a slight impact on the Glide scores (results not shown). Thus, to avoid an improperly folded loop affecting the scoring of the protein-ligand complexes, the C-terminal part of EL2 (residues P620-E656) was deleted from the post-IFD cluster 1 mGlu5-based models.

Following IFD, each cluster of PAMs and decoys were docked into the 456 IFD-optimised models and the docking results were analysed using the BEDROC method as described above. The results of the BEDROC analysis showed that IFD significantly improved the docking results and no further optimisation of the complexes were thus performed.

#### Selection of final models

To select GABA_B2_ homology models from each cluster of actives, the 456 IFD-optimised models were ranked according to their BEDROC scores. Visual inspection of the PAM docking results in the 10 best ranked models or with BEDROC scores > 0.5 was performed by assessing the location of the PAMs in the binding pocket and the constancy of the orientations of the actives within each cluster of PAMs (S4 Table). Due to the low BEDROC scores obtained (BEDROC < 0.5; S4 Table), none of the models based on docking of the cluster 3 PAMs were included. Thus, eight final models, two models per cluster 1, 2, 4, and 5 PAMs, were selected.

#### SIFt analysis

Structural Interaction Fingerprints (SIFt) were used to map the allosteric binding site of GABA_B2_ subunit in the eight selected homology models [46,47]. The residues around each docked ligand and the type of interaction was determined based on distance (cut-off 4Å) and atom/residue type (and angle in case of hydrogen bonds), and for every accepted ligand-residue interaction, the appropriate fingerprint bits (any contact, backbone, side chain, polar, hydrophobic, hydrogen bond donor, hydrogen bond acceptor, aromatic, and/or charged) were turned on [47]. A SIFt is hence a binary pattern describing interactions between all amino acids in contact with a ligand. SIFts describing the interactions of individual ligands in the GABA_B_ allosteric binding pocket were averaged into SIFt profiles based on different input taking only the 'any contact' bit into account and applying a cutoff of 50% (default settings) [47]. Two general 2D-SIFT profiles, SIFt(8) and SIFt(7), were constructed. While SIFt(8) was constructed based on the docking results of the PAMs in clusters 1, 2, 4, and 5 in their respective models (mGlu- and rhodopsin-based models), SIFt(7) was based solely on the docking results of the cluster 1, 2, 4, and 5 PAMs in the seven mGlu-based models. In addition, three template-specific 2D-SIFt profiles were generated based on docking results in the rhodopsin-based model (input: one model—cluster 1 PAMs, SIFt(1U19)), mGlu1-based models (input: two models—cluster 4 PAMs, SIFt(4OR2)), and mGlu5-based models (input: five models—cluster 1, 2, and 5 PAMs, SIFt(4OO9)). Finally, three cluster-specific 2D-SIFt profiles were constructed based on the docking of the PAMs in clusters 1 (input: one rhodopsin- and one mGlu5-based model, SIFt(C1)), cluster 2 (input: two mGlu5-based models, SIFt(C2)), and cluster 5 (two mGlu5-based models, SIFt(C5)). SIFt(C4) was generated based on cluster 4 PAMs and was hence identical with SIFt(4OR2).

### GPCR family C numbering scheme

The GPCR database class C (GPCRdb(C)) numbering scheme [48] is used in the paper to facilitate comparison between class C receptors. A sequence-based scheme, where the first digit refers to the TM helix, the second digit to the position of the amino acid in relation to the most conserved amino acid of the helix and the third digit, separated by x, corrects for helical bulges and constrictions [48], is used. The amino acid in position 3.50x50, for instance, is the most conserved amino acid in TM3.

## Results

In the present study, homology models of the GABA_B2_ subunit containing the allosteric binding pocket of the GABA_B_ receptor have been constructed using x-ray crystal structure templates from GPCR classes A, B, and C. The results of the amino acid sequence alignment of templates and target showed that the sequence identity between the 7TM domain GABA_B2_ subunit and the class A and B crystal structures was ranging between 10% and 13% but increased significantly between GABA_B2_ subunit and the class C members mGlu1 and mGlu5 (19% and 22%, respectively) (S1 Fig). The 7TM sequence similarity between GABA_B2_ subunit and the class A and B templates were ranging from 28% to 34%, and more than 40% for the class C templates (S1 Fig).

Through iterative steps of homology modelling and docking analysis of five clusters of PAMs vs. decoys, the number of GABA_B2_ homology models was reduced from the initial 600 models, via 456 induced-fit models, to eight final GABA_B2_ homology models (S5 Table). These models are available as supporting information (S1–S8 Models). The results showed that seven of the eight models originated from class C x-ray crystal structure templates (two mGlu1- and five mGlu5-based models), while the last model was constructed based on rhodopsin template (S5 Table). The GABA_B2_ models based on the class A β2-AR (PDB id 2RH1) and class B CRF1 receptor (PDB id 4K5Y) were outperformed by the models based on rhodopsin and the mGlu receptors in the docking of PAMs versus decoys (S4 Table). For GABA_B2_ homology models based on the class B human glucagon receptor (PDB id 4L6R), no representative orientations of the PAMs could be selected and no IFD optimisation was hence performed.

The ability of the constructed models to enrich positive allosteric modulators among significantly higher numbers of decoys when docked into the putative allosteric binding site was used for selection of eight final GABA_B2_ homology models. The eight final GABA_B2_ models were chosen by selecting two models based the docking of each cluster of PAMs except the cluster 3 PAMs (S4 Table). Analysis of the docking results of cluster 3 PAMs showed that the best enrichment of the active ligands were in GABA_B2_ homology models constructed based on class B CRF1 receptor (PDB id 4K5Y) (S4 Table), though no models were selected due to the low BEDROC scores (< 0.5; S4 Table). However, all 13 cluster 3 PAMs docked into the two selected mGlu1-based models and one of the selected mGlu5-based model (model C2_M1_4OO9), though the scores of the cluster 3 PAMs in these models (and hence the BEDROC values) were not as good as the scores of the cluster 4 and 2 PAMs docked into these models, respectively (S5 Table). Visual inspection of the docking results showed that the best orientations of the cluster 3 PAMs were in the mGlu1-based models, but more variation in the binding orientations of the ligands were seen than for the cluster 4 PAMs in these models (results not shown). The cluster 3 PAMs also docked in the rhodopsin-based model; however, visual inspection of the results showed that multiple of the ligands were docked outside the binding site.

**Fig 4:**
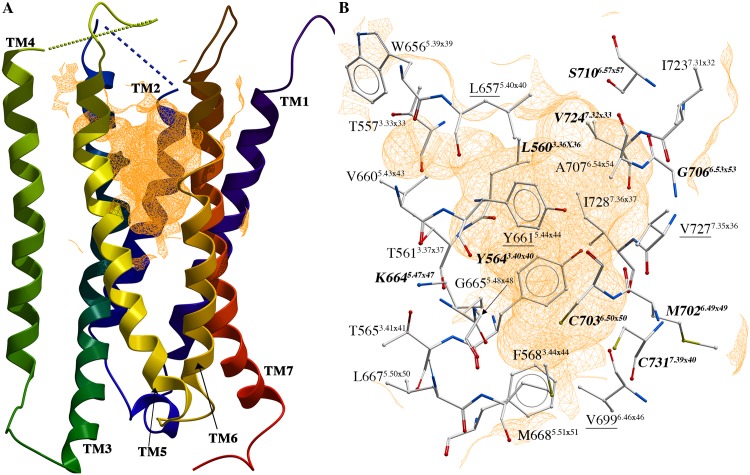
Putative GABA_B2_ allosteric binding pocket. (A) The 7TM domain of model c1_m2_4009 (mGlu5-based model) with putative allosteric binding site shown as orange mesh. (B) Location of the allosteric binding site identified through docking of PAMs shown in model c1_m2_4009 (mGlu5-based model). Hotspot residues shown in bold/italics (SIFt(8) hotspots) and bold/italics/underlined (SIFt(7) hotspots) (Table 1). The figure has been generated using ICM software version 3.8–0 [49].

To map the allosteric binding site of GABA_B2_ subunit based on the docking results of the PAMs, structural interaction fingerprint (SIFt) profiles were generated (Table 1, S6 Table. Fig 4). The general SIFt profile (SIFt(8)) revealed that 24 amino acids in TMs 3, 5, 6, and 7 were within 4 Å of more than 50% of the ligands in clusters 1, 2, 4, and 5 PAMs in the eight GABA_B2_ models (Table 1), while one additional residue was identified in the SIFt(7) general profile constructed using only the mGlu-based models (Table 1). Of the 24 SIFt(8) amino acids, nine 'hotspot' residues (defined as residues with SIFt(8) values ≥ 0.95) were identified, while four additional hotspots were observed when excluding the docking results in the rhodopsin-based models from the analysis (SIFt(7), Table 1). The results also showed that the GABA_B2_ allosteric binding site was hydrophobic in nature; only ten of the putative allosteric binding site amino acids were polar or charged, the majority of which were located in TMs 3 and 5 (Table 1).

**Table 1:** Putative GABA_B_2 allosteric binding site. SIFt(8), SIFt(7) profiles and corresponding amino acids in GABA_B2_ subunit and mGlu1 and mGlu5 receptors. GPCRdb(C), GPCR database numbering. Amino acids in bold have been shown to change binding/potency of allosteric ligands > 5-fold when mutated in other class C human receptors (data extracted from GPCRdb [5,6] accessed Nov. 25, 2015).

GPCRdb(C)	GABA_B2_	mGlu_1_	mGlu_5_	SIFt(8)	SIFt(7)
**3.33x33**	**T557**	**R661**	**R648**	**0.67**	**0.74**
**3.36x36**	**L560**	**V664**	**I651**	**1**	**1**
**3.37x37**	**T561**	**G665**	**G652**	**0.7**	**0.67**
**3.40x40**	**Y564**	**S668**	**P655**	**1**	**1**
3.41x41	T565	A669	A656	0.62	0.58
**3.44x44**	**F568**	**Y672**	**Y659**	**0.71**	**0.79**
5.39x39	W656	G752	G739	0.6	0.56
**5.40x40**	**L657**	**V753**	**V740**	**0.9**	**1**
5.43x43	V660	P756	P743	0.63	0.68
**5.44x44**	**Y661**	**L757**	**L744**	**0.9**	**1**
**5.47x47**	**K664**	**N760**	**N747**	**0.99**	**1**
5.48x48	G665	G761	G748	0.57	0.63
5.50x50	L667	L763	L750	0.46	0.52
5.51x51	M668	I764	I751	0.75	0.83
6.46x46	V699	T794	T781	0.9	1
6.49x49	M702	I797	I784	1	1
**6.50x50**	**C703**	**W798**	**W785**	**1**	**1**
**6.53x53**	**G706**	**F801**	**F788**	**0.97**	**0.96**
6.54x54	A707	V802	V789	0.9	0.88
**6.57x57**	**S710**	**Y805**	**Y792**	**1**	**1**
7.31x32	I723	T814	T801	0.68	0.75
**7.32x33**	**V724**	**T815**	**M802**	**1**	**1**
7.35x36	V727	A818	S805	0.9	1
**7.36x37**	**I728**	**V819**	**V806**	**0.69**	**0.65**
**7.39x40**	**C731**	**S822**	**S809**	**0.98**	**1**

**Fig 5:**
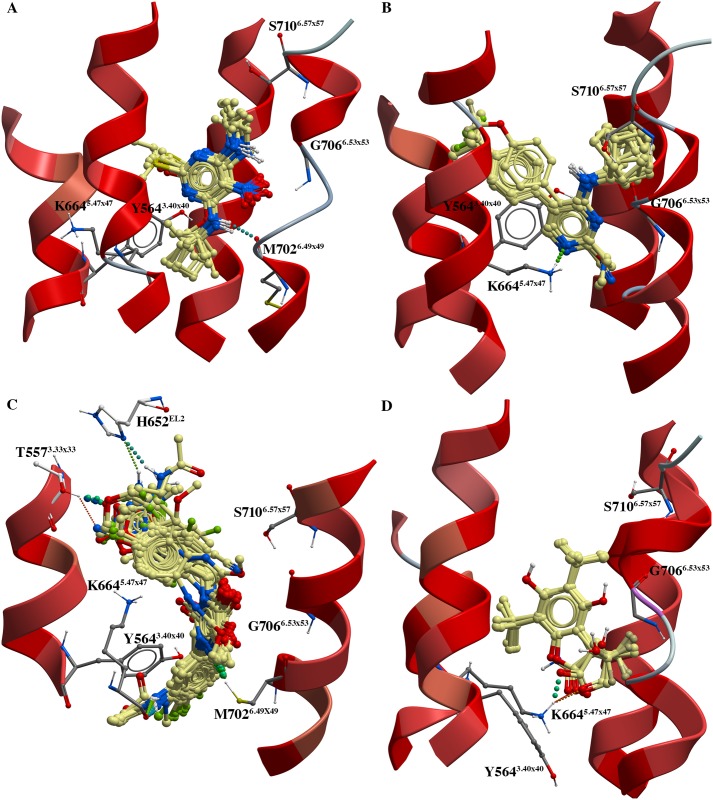
PAM docking poses. (A) Cluster 1 PAMs in model c1_m2_4OO9 (mGlu5-based model), (B) cluster 2 PAMs in model c2_m1_4OO9 (mGlu5-based model), (C) cluster 4 PAMs in model c4_m2_4OR2 (mGlu1-based model), and (D) cluster 5 PAMs in model c5_m1_4OO9 (mGlu5-based model). Intermolecular hydrogen bonds shown as dotted lines. Images generated using ICM software version 3.8–0 [49].

The models selected based on the docking of the cluster 1 PAMs were the only models selected that had been constructed based on different templates, namely the class A rhodopsin (PDB id 1U19) and class C mGlu5 receptor (PDB id 4OO9). In comparison to the mGlu-based models, the ligands docked closer to the extracellular environment in the rhodopsin-based model (Fig 5, S2 Fig). In the rhodopsin-based model, the side chain of N650^EL2^ formed hydrogen bonds to the–NO_2_ moieties of the cluster 1 PAMs and the side chain of S710^6.57x57^ was in hydrogen bonding distance of the protonated nitrogen ligand moieties (S2 Fig). In comparison, possible hydrogen bonds between the ligand–NO_2_ moieties and/or protonated nitrogen moieties and M702^6.49x49^ and G706^6.53x53^ backbone atoms, located deeper in the allosteric binding pocket, were observed in the mGlu5-based model (Fig 5).

Though the 3D structures of the models were more conserved among the seven mGlu-based models than between the rhodopsin- and mGlu-based models, significant differences were also observed between the mGlu-based GABA_B2_ models. Superposition of the models revealed minor horizontal shifts in the TMs around the putative allosteric binding site, especially in TMs 5, 6 and 7 (S3 Fig). TM6 was moreover partly unwound in the allosteric binding site region in the mGlu5-based models selected based on docking of cluster 1 and 5 PAMs (Fig 5, S2 and S3 Figs). Several significant differences were also observed in the orientations of the amino acids constituting the putative allosteric binding site of the mGlu-based GABA_B2_ models (Fig 5, S2 Fig). In particular, the orientations of the side chains of Y564^3.40x40^ and K664^5.47x47^ varied significantly between the models, and in some models, the K664^5.47x47^ charged side chain pointed away from the ligands in the binding site (Fig 5, S2 Fig). Differences in the location of TM6 amino acids in the putative allosteric binding site were also observed, mainly reflecting differences in unwinding and/or horizontal shifts in TM6 in this region (Fig 5, S2 and S3 Figs). However, visual inspection of the docking results also showed that commonly observed interactions between the PAMs and the amino acids of the GABA_B2_ allosteric binding site were perpendicular and/or sandwich stacking interactions between the ligands and the aromatic side chain of Y564^3.40x40^ and/or Y661^5.44x44^ (Fig 5, S2 Fig). Backbone and/or side chain interactions with amino acids K664^5.47x47^ and M702^6.49x49^, C703^6.50x50^, and G706^6.53x53^ were also observed with multiple of the docked PAMs, while hydrophobic interactions with amino acids in all four TMs contributing to the allosteric binding site were also common with all PAMs (Fig 5, S2 Fig).

## Discussion

Within the last 10 years, critical advances in the experimental characterisation of GPCRs have led to the determination of multiple class A receptors in different activation states [50] and the first structure bound to a G-protein [51]. In addition, the first structures of class B, C, and F members were recently published [31,52–55]. The majority of the currently available structures are of class A GPCRs, and it has been estimated that more than 60% of the members of class A can be predicted using the homology modelling technique given a minimum of 30% TM sequence identity with one of the available crystal structures [56]. Assessment of the accuracy of homology modelling of membrane proteins has indicated that a sequence identity of approx. 30% may yield a transmembrane region Cα-RMSD to the native of 2 Å or less, provided that an accurate template-target sequence alignment can be achieved [57]. The importance of template-target sequence alignment on the quality of homology models was also recently highlighted in the GPCR dock 2013 assessment [58]. The class F smoothened (SMO) receptor shared a sequence identity of approx. 14% with the closest template available and the GPCR dock 2013 results showed that the median 7TM and binding pocket RMSD for the SMO receptor in complex with the ligand SANT-1 were 6.33 Å and 10.66 Å, respectively [58]. Hence, despite the recent releases of x-ray crystal structures of more closely related GPCRs to the GABA_B_ receptor, homology modelling of the GABA_B2_ subunit was still highly challenging. In the current study, the template-target alignments were adjusted based on structural information from six x-ray crystal structure templates. The overall 7TM sequence identity between GABA_B2_ subunit and its closest homologues, the mGlu1 and mGlu5 receptors, was approx. 20%, significantly higher than for the class A and B templates (approx. 10%-13%) (S1 Fig). The results of this study also unambiguously show that GABA_B2_ homology models constructed using class C templates were the best to enrich the known PAMs–seven of eight final models were based on the two class C templates.

To predict the 3D structure of the GABA_B2_ TM domain, MODELLER software [33] was chosen to scan the conformational space of GABA_B2_ subunit and to decrease the ligand bias inherited from the selected templates. The ability of the models to enrich known GABA_B_ receptor PAMs that had been grouped into 5 diverse clusters based on ligand structure (S1 Table) among significantly higher number of decoys was used to guide the selection of GABA_B2_ homology models. Different conformations of the GABA_B2_ subunit specific for each cluster of PAM were thus obtained. Initially, docking was performed into the 600 crude GABA_B2_ models (i.e., 100 models/template), followed by optimisation of the models using the induced-fit docking approach in which rigid receptor docking was combined with protein structure prediction and refinement [59], and redocking of the PAM-decoy sets. The ligand-guided homology modelling approach hence combined IFD and docking of actives vs. decoys (virtual ligand screening enrichment), approaches that have successfully been applied to generate high-quality homology models of other GPCRs [60]. The approach is also in line with multiple other studies showing the importance of incorporating ligand information and experimental knowledge into homology modelling protocols. Different approaches have previously successfully been used, ranging from receptor-ligand restraints to different IFD and ligand-steered docking protocols [61–64].

A challenge of the ligand-guided homology modelling of GABA_B2_ subunit was, however, the relatively low number and limited structural diversity of the currently known GABA_B_ allosteric modulators (S1 Table). The activities of the available PAMs had, moreover, been evaluated using different types of in vitro functional assays and/or using different concentrations of GABA (10 nM, 1000 nM, 10.000 nM, not always given in the literature) (S1 Table). EC_50_ values were not available for all modulators (S1 Table), and in case of the cluster 4 PAMs, all ligands except ADX71943 were retrieved from patent literature and only an EC_50_ range of each PAM was hence available (< 100 nM, 100–500 nM, 500–1000 nM) (S1 Table).

Site-directed mutagenesis data can be highly useful when evaluating homology models and docking results. For the GABA_B_ receptor, only one site-directed mutagenesis study that describes the effects of mutations of amino acids of the 7TM domain of the GABA_B2_ subunit from rat has been published [14]. However, mutations of several of the putative allosteric binding site residues identified through docking of the PAMs in the present study (Table 1) was shown to affect the ability of the positive modulator GS39783 (cluster 1) to activate the rat GABA_B_ receptor [14]. The site-directed mutagenesis data, for instance, showed that the rat GABA_B2_ G706T^6.53x53^/A708P^6.55x55^/S710T^6.57x57^ triple mutant conferred agonistic activity to GS39783 (cluster 1 PAM) and CGP7930 (cluster 5 PAM) in absence of GABA [14]. In the present study, G706^6.53x53^ and S710^6.57x57^ were within 4 Å of the majority of the PAMs in all eight final models (Table 1), while A708^6.55x55^ pointed away from the binding pocket (helical turn) and was not identified as being part of the allosteric binding pocket. Comparison of the mGlu-based GABA_B2_ homology models revealed a horizontal movement of the extracellular part of TM6 (S3 Fig) and showed that the helix was partly unwound in the region around G706^6.53x53^ in the mGlu5-based GABA_B2_ models selected through docking of cluster 1 and 5 PAMs (Fig 5, S2 Fig). Glycine and proline residues are often found in TM regions that are unwound or contain helical kinks, and movement of TM6 of GPCRs is known to play a pivotal role during activation of the GPCRs [65]. The present results in combination with the rat GABA_B2_ site-directed mutational data may thus indicate that the switch from positive modulation to agonism of GS39783 and CGP7930 induced by the G706T^6.53x53^ and A708P^6.55x55^ mutations may be the results of changes in the flexibility/movements of the extracellular part of TM6.

The docking results also indicated that hydrogen bonds between the -NO_2_ moiety of GS39783 and other cluster 1 PAMs and the backbone of G706^6.53x53^ may be formed (Fig 5, S2 Fig). Due to the structural differences between GS39783 (cluster 1 PAM) and CGP7930 (cluster 5 PAM) (Fig 3, S1 Table), the docking results in contrast suggested that interactions with K664^5.47x47^ (currently not mutated in GABA_B_ receptor) may be more important for binding of the CGP7930 and the other cluster 3 PAMs than hydrogen bonding interactions with TM6 residues (Fig 5, S2 Fig). This may provide an explanation to why the obtained agonistic efficacy of CGP7930 in the G706T^6.53x53^/A708P^6.55x55^/S710T^6.57x57^ and G706T^6.53x53^/A708P^6.55x55^ rat GABA_B2_ mutants, was only approx. 25% of the response obtained with GS39783 [14]. Interestingly, the docking results also showed that a hydrophobic ligand moiety of PAMs in clusters 1 and 5 were located in the vicinity of S710^6.57x57^ (Fig 5, S2 Fig), providing an explanation to why the S710T^6.57x57^ mutation was not necessary to confer agonistic activity of the two PAMs [14].

Considerably more site-directed mutagenesis data is available from studies on the other class C members than GABA_B_ receptor. Though extrapolation of mutagenesis data from other class C members is extremely difficult due to the very low sequence identity between the class members, it is clear that mutations of amino acids at certain positions in the TM helices give significant changes in ligand binding or potency across multiple class C members [5,6]. Mutations that result in > 5-fold changes in ligand binding/potency in one or more class C members are highlighted in Table 1. Of the nine hotspot amino acids identified by docking of the PAMs into the eight GABA_B2_ models based on the mGlu and rhodopsin templates (Fig 4, Table 1), mutations of amino acids in the corresponding positions have been shown to give significant changes in other class C GPCRs except for position 6.49x49—corresponding to M702^6.49x49^ in GABA_B2_ subunit (Table 1). In case of the four additional hotspots from SIFt(7) analysis, residues in two of the positions, L657^5.40x40^ and Y661^5.44x44^, have been mutated and shown to significantly affect ligand potency in other class C GPCRs, while V699^6.46x46^ and V727^7.35x36^ have not (Table 1).

Though the overall sequence identity may be relatively low between GPCRs, the sequence identity is usually significantly higher in the orthosteric binding sites of the receptors. From a homology modelling perspective, higher sequence identity is positive as the quality of the models in binding site region thus usually improves. From a pharmacological point of view, however, the increased conservation of the orthosteric binding pocket is challenging as it makes development of selective ligands more difficult, and this is one of the major reasons why development of allosteric modulators is highly interesting. In line with this, comparison between the 24 (25) amino acids identified through docking of known PAMs to constitute the putative GABA_B_ receptor allosteric binding site and the corresponding residues in mGlu1 and mGlu5 receptors showed little sequence identity, though the overall sequence similarities were significantly higher (33% and 38% for mGlu1 and mGlu5, respectively) (Table 1). Comparison of the GABA_B2_ (Table 1) and mGlu 1–8 receptor allosteric binding sites as reported by Doré et al. [43] highlighted several similarities and differences between the receptors. In total, 16 common amino acid positions were identified between the GABA_B2_ and mGlu 1–8 receptor allosteric binding sites (Table 1, [43]). Amino acids in several of these positions shared common characteristics; hydrophobic amino acids were found corresponding to L560^3.36x36^, L667^5.50x50^, M668^5.51x51^, M702^6.49x49^, V724^7.32x33^ (except T/mGlu1), and I728^7.36x37^, while the amino acids in positions F568^3.44x44^ and C731^7.39x40^ were aromatic and polar, respectively (Table 1, [43]). In contrast, the amino acids corresponding to T561^3.37x37^, Y661^5.44x44^, V699^6.46x46^, C703^6.50x50^, G706^6.53x53^, and V727^7.35x36^ were highly conserved between the mGlu 1–8 receptors but were non-conserved between the mGlu receptors and GABA_B2_, whereas the amino acids corresponding to Y564^3.40x40^, V660^5.43x43^, and K664^5.47x47^ varied among all nine receptors (Table 1, [43]). Interestingly, multiple of the amino acids identified in the current work to be important for binding of the PAMs to GABA_B2_ subunit, were located in these variable positions—including Y564^3.40x40^ (P, S, F, or T in mGlu receptors) and Y661^5.44x44^ (L in all mGlu receptors), K664^5.47x47^ (N, D, or S in mGlu receptors), C703^6.50x50^ (W in all mGlu receptors), and G706^6.53x53^ (F in all mGlu receptors). In addition, one of the identified SIFt(8) hotspot amino acid positions, S710^6.57x57^, was not part of the mGlu receptor allosteric binding site [43]. The corresponding amino acids in this position in the mGlu receptors are aromatic in nature [43]. Likewise, the residue in the position corresponding to the SIFt(7) hotspot L657, 5.40x40, was not included in the mGlu receptor allosteric binding sites [43]. The results of this study hence indicate that design of selective GABA_B_ receptor allosteric modulators is possible.

## Conclusions

In the present study, the first atomic-resolution description of the GABA_B2_ putative allosteric binding site in complex with positive allosteric modulators have been determined through homology modelling and docking of positive allosteric modulators. Analysis of the docking results and comparison with mutagenesis data from GABA_B_ and other human class C GPCRs highlight several interesting differences between the homologous receptors and suggest several residues that may infer ligand selectivity. The eight GABA_B_ receptor homology models generated moreover enriched the known active PAMs before presumably inactive compounds and may hence be useful tools in virtual screening campaigns to discover new allosteric modulators. Despite no models being selected based on the docking of cluster 3 PAMs, these PAMs also docked into the two mGlu1-based models selected through docking of the cluster 4 PAMs and one of the cluster 2 mGlu5-based models, indicating that the current eight models also may be used to identify analogues of cluster 3 PAMs in virtual screening campaigns. The eight models constructed in the present study may thus aid the discovery of novel GABA_B_ receptor allosteric modulators through screening and the structure-based design and optimisation of GABA_B_ allosteric modulators.

## Supporting information

S1 Fig 7TM alignments of GABA_B2_ and template sequences. 1U19_A, rhodopsin chain A; 2RH1_A, β_2_-AR chain A; 4K5Y_C, CRF1R chain C; 4L6R_A, glucagon receptor chain A; 4OO9_A_A, mGlu5 receptor chain A; 4OR2_B, mGlu1 receptor chain B. The 7TM sequence identity between GABA_B2_ and templates is shown.
(PDF)

S2 Fig PAM docking poses. a) Cluster 1 ligands in model C1_M1_1U19 (rhodopsin-based model), b) cluster 2 ligands in model C2_M2_4OO9 (mGlu5-based model), c) cluster 4 ligands in model C4_M1_4OR2 (mGlu1-based model), d) cluster 5 ligands in model C5_M2_4OO9 (mGlu5-based model). Intermolecular hydrogen bonds shown as dotted lines. Images generated using ICM software version 3.8–0 [49].
(PDF)

S3 Fig Superposition of GABAB2 homology models. Superimposition of models constructed based on the mGlu1 (PDB id 4OR2) and mGlu5 (PDB id 4OO9) templates. horizontal shift in TM6 (G7006.47x47-S7106.57x57) in the models highlighted in grey. Images generated using ICM software version 3.8–0 [49].
(PDF)

S1 Model Model c1_m1_1u19.pdb. Cluster 1 based model 1. Template 1u19.
(PDB)

S2 Model Model c1_m2_4oo9.pdb. Cluster 1 based model 2. Template 4oo9.
(PDB)

S3 Model Model c2_m1_4oo9.pdb. Cluster 2 based model 1. Template 4oo9.
(PDB)

S4 Model Model c2_m2_4oo9.pdb. Cluster 2 based model 2. Template 4oo9.
(PDB)

S5 Model Model c4_m1_4or2.pdb. Cluster 4 based model 1. Template 4or2.
(PDB)

S6 Model Model c4_m2_4or2.pdb. Cluster 4 based model 2. Template 4or2.
(PDB)

S7 Model Model c5_m1_4oo9.pdb. Cluster 5 based model 1. Template 4oo9.
(PDB)

S8 Model Model c5_m2_4oo9.pdb. Cluster 5 based model 2. Template 4oo9.
(PDB)

S1 Table Cluster 1–5 PAMs. Ligand structures, names, biological data, and sources are given. G, GABA: B, Baclofen: MW, molecular weight (g/mol).
(PDF)

S2 Table Structures of experimentally known inactive ligands used as decoys. (PDF)

S3 Table Induced-Fit Docking (IFD) input ligands. See S1 Table for ligand activity data and structures.
(PDF)

S4 Table BEDROC values of 10 best models per ligand cluster. 8 final models highlighted in red. 1U19, rhodopsin-based models; 4OR2, mGlu1-based models; 4OO9, mGlu5-based models; 4K5Y, CRF1R-based models; 2RH1, β_2_-AR-based models.
(PDF)

S5 Table BEDROC values and Glide score ranges from docking of cluster 1–5 PAMs into the 8 final GABA_B2_ homology models. (PDF)

S6 Table SIFt profiles. Amino acids within 4 Å of more than 50% of the ligands are listed. SIFt(8), SIFt(7) general SIFt profiles; SIFt(C1-C5), cluster-specific SIFt profiles; SIFt(1U19-4OR2-4OO9), template-specific SIFt profiles. GPCRdb (C), class C GPCR database numbering scheme positions.
(PDF)
