# Supplementary material for: Ligand-guided homology modelling of the GABAB2 subunit of the GABAB receptor
Source: PLoS One. 2017 Mar 21;12(3):e0173889. doi: 10.1371/journal.pone.0173889 (PMC5360267; doi:10.1371/journal.pone.0173889)
Supplement: S1 Fig — 1U19_A, rhodopsin chain A; 2RH1_A, β2-AR chain A; 4K5Y_C, CRF1R chain C; 4L6R_A, glucagon receptor chain A; 4OO9_A_A, mGlu5 receptor chain A; 4OR2_B, mGlu1 receptor chain B. The 7TM sequence identity between GABAB2 and templates is shown. (PDF) [file pone.0173889.s001.pdf]

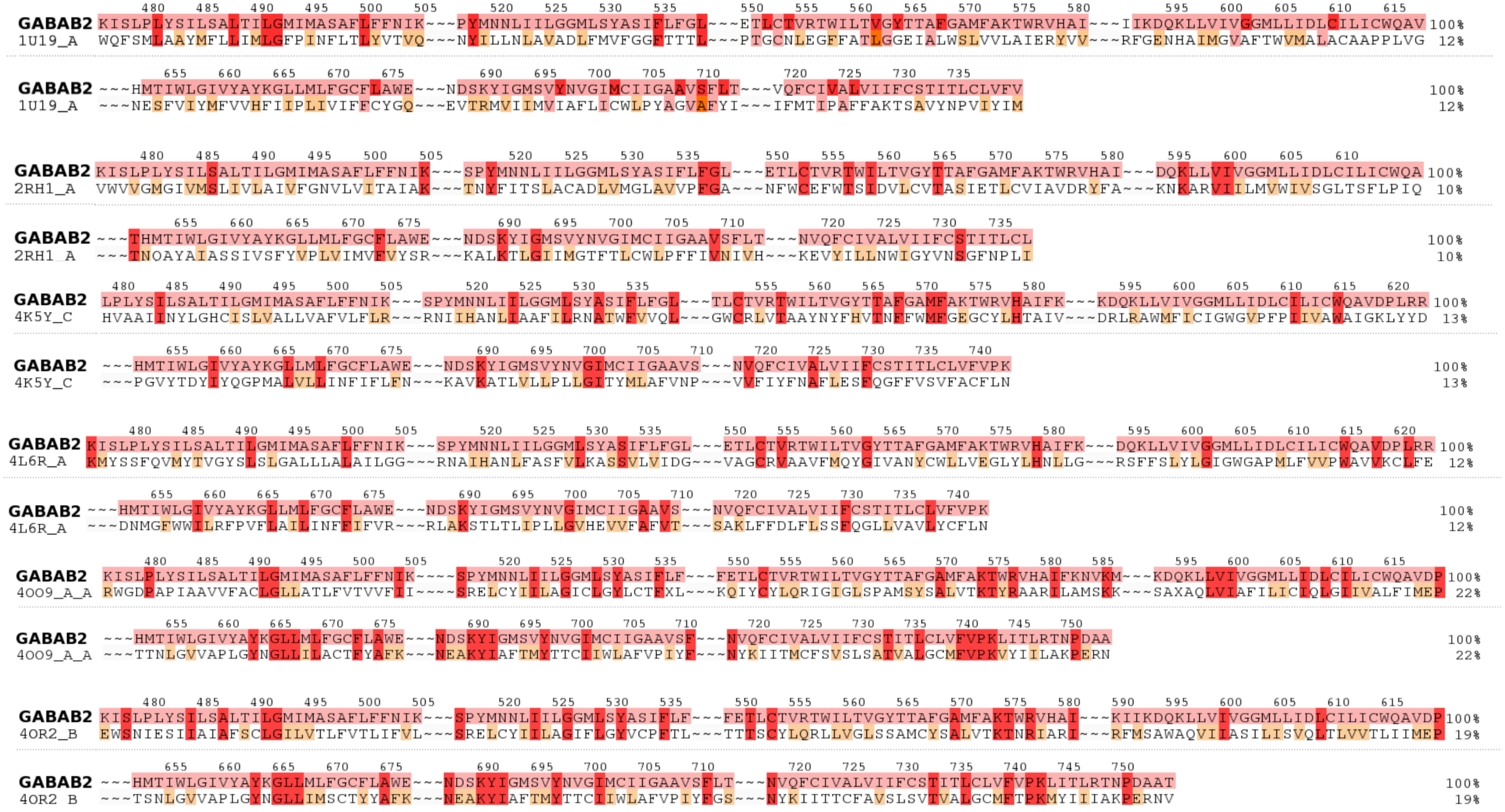

S1 Fig. 7TM alignments of GABA$_{B2}$ and template sequences. 1U19_A, rhodopsin chain A; 2RH1_A, β_2-AR chain A; 4K5Y_C, CRF1R chain C; 4L6R_A, glucagon receptor chain A; 4OO9_A_A, mGlu5 receptor chain A; 4OR2_B, mGlu1 receptor chain B. The 7TM sequence identity between GABA$_{B2}$ and templates is shown.
