# Supplementary material for: Ligand-guided homology modelling of the GABAB2 subunit of the GABAB receptor
Source: PLoS One. 2017 Mar 21;12(3):e0173889. doi: 10.1371/journal.pone.0173889 (PMC5360267; doi:10.1371/journal.pone.0173889)
Supplement: S2 Fig — a) Cluster 1 ligands in model C1_M1_1U19 (rhodopsin-based model), b) cluster 2 ligands in model C2_M2_4OO9 (mGlu5-based model), c) cluster 4 ligands in model C4_M1_4OR2 (mGlu1-based model), d) cluster 5 ligands in model C5_M2_4OO9 (mGlu5-based model). Intermolecular hydrogen bonds shown as dotted lines. Images generated using ICM software version 3.8–0 [49]. (PDF) [file pone.0173889.s002.pdf]

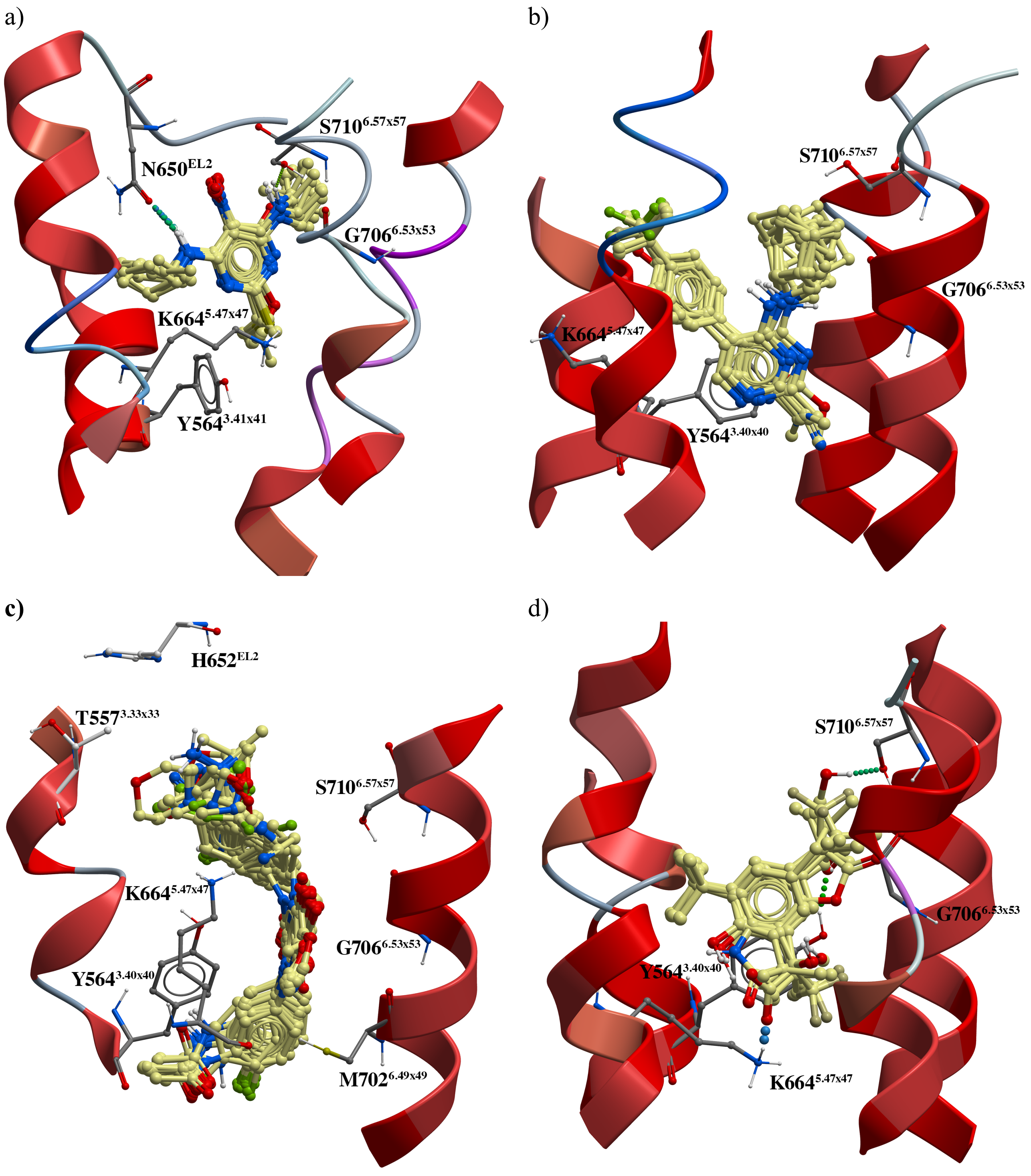

S2 Fig. **PAM docking orientations**. a) Cluster 1 ligands in model C1_M1_1U19 (rhodopsin-based model), b) cluster 2 ligands in model C2_M2_4OO9 (mGlu5-based model), c) cluster 4 ligands in model C4_M1_4OR2 (mGlu1-based model), d) cluster 5 ligands in model C5_M2_4OO9 (mGlu5-based model). Intermolecular hydrogen bonds shown as dotted lines. Images generated using ICM software version 3.8-0 (Abagyan and Totrov 1994).
