# Supplementary material for: Ligand-guided homology modelling of the GABAB2 subunit of the GABAB receptor
Source: PLoS One. 2017 Mar 21;12(3):e0173889. doi: 10.1371/journal.pone.0173889 (PMC5360267; doi:10.1371/journal.pone.0173889)

S3 Fig. Superposition of GABA$_{B2}$ homology models. Superimposition of models constructed based on the mGlu1 (PDB id 4OR2) and mGlu5 (PDB id 4OO9) templates. horizontal shift in TM6 (G700$^{6.47x47}$-S710$^{6.57x57}$) in the models highlighted in grey. Images generated using ICM software version 3.8-0 (Abagyan and Totrov 1994)

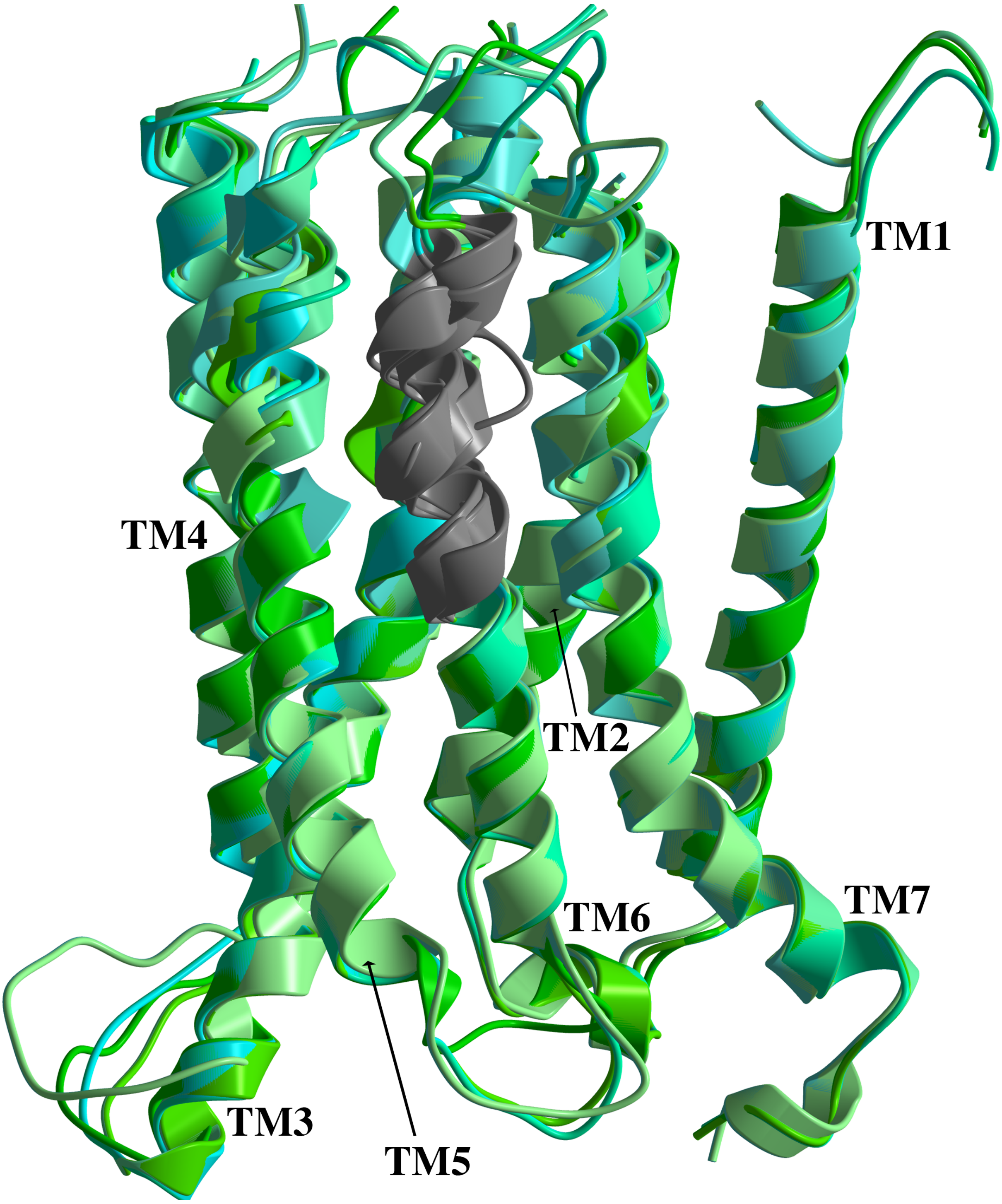

Supplement: S3 Fig — Superimposition of models constructed based on the mGlu1 (PDB id 4OR2) and mGlu5 (PDB id 4OO9) templates. horizontal shift in TM6 (G7006.47x47-S7106.57x57) in the models highlighted in grey. Images generated using ICM software version 3.8–0 [49]. (PDF) [file pone.0173889.s003.pdf]
