# Supplementary material for: Ligand-guided homology modelling of the GABAB2 subunit of the GABAB receptor
Source: PLoS One. 2017 Mar 21;12(3):e0173889. doi: 10.1371/journal.pone.0173889 (PMC5360267; doi:10.1371/journal.pone.0173889)
Supplement: S1 Table — Ligand structures, names, biological data, and sources are given. G, GABA: B, Baclofen: MW, molecular weight (g/mol). (PDF) [file pone.0173889.s012.pdf]

S1 Table. Cluster 1-5 PAMs. Ligand structures, names, biological data, and sources are given. G, GABA: B, Baclofen: MW, molecular weight (g/mol).

Structure	Name in litterature	Biolocical Data				MW g/mol	Source
		Experiment	Activity type	Activity value	[G] / [B] nM		
**Cluster1**							
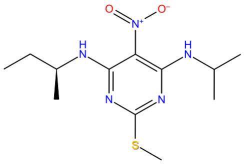	CGP10773	[^{35}S]-GTPγS binding	Emax % [10uM]	78	1000 (G)	299	Urwyler,2003
		[^{35}S]-GTPγS binding	Emax % [1uM]	40	1000 (G)	299	Urwyler,2003
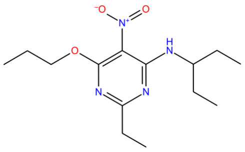	CGA50739	[^{35}S]-GTPγS binding	Emax % [1uM]	42	1000 (G)	296	Urwyler,2003
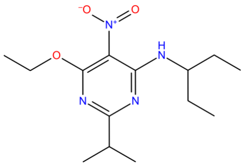	CGA44010	[^{35}S]-GTPγS binding	Emax % [1uM]	20	1000 (G)	296	Urwyler,2003
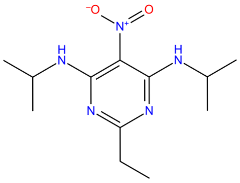	CGA38906	[^{35}S]-GTPγS binding	Emax % [1uM]	34	1000 (G)	267	Urwyler,2003
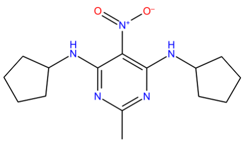	CGA38493	[^{35}S]-GTPγS binding	Emax % [1uM]	48	1000 (G)	305	Urwyler,2003
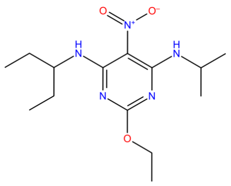	CGA37456	[^{35}S]-GTPγS binding	Emax % [1uM]	38	1000 (G)	311	Urwyler,2003

Structure	Name in litterature	Biolocical Data				MW g/mol	Source
		Experiment	Activity type	Activity value	[G] / [B] nM		
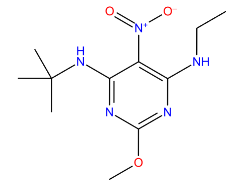	CGA28942	[^{35}S]-GTPγS binding	Emax % [1uM]	30	1000 (G)	269	Urwyler,2003
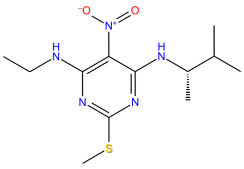	CGA19418	[^{35}S]-GTPγS binding	Emax % [1uM]	33	1000 (G)	299	Urwyler,2003
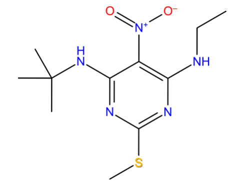	CGA14566	[^{35}S]-GTPγS binding	Emax % [1uM]	35	1000 (G)	285	Urwyler,2003
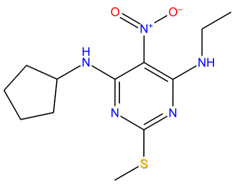	CGA19414	[^{35}S]-GTPγS binding	Emax % [1uM]	43	1000 (G)	297	Urwyler,2003
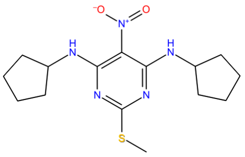	GS39783	[^{35}S]-GTPγS binding	EC$_{50}$ [nM]	3100	1000 (G)	337	Urwyler,2003
**Cluster2**							
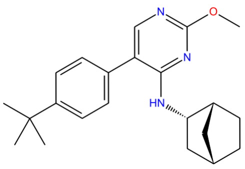	41	[^{35}S]-GTPγS binding	EC$_{50}$ [nM]	2040	1000 (G)	351	Guery,2007
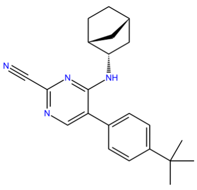	40	[^{35}S]-GTPγS binding	EC$_{50}$ [nM]	1202	1000 (G)	346	Guery,2007

Structure	Name in litterature	Biolocical Data				MW g/mol	Source
		Experiment	Activity type	Activity value	[G] / [B] nM		
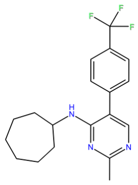	29	[^{35}S]-GTPγS binding	EC_{50} [nM]	1660	1000 (G)	349	Guery,2007
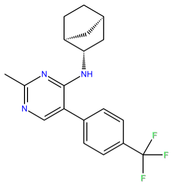	27 (BHF177)	[^{35}S]-GTPγS binding	EC_{50} [nM]	1660	1000 (G)	347	Guery,2007
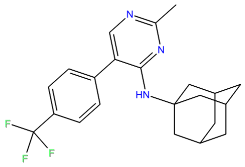	26	[^{35}S]-GTPγS binding	EC_{50} [nM]	3467	1000 (G)	387	Guery,2007
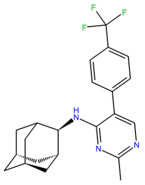	25	[^{35}S]-GTPγS binding	EC_{50} [nM]	2754	1000 (G)	387	Guery,2007
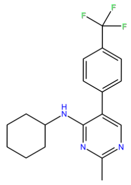	22	[^{35}S]-GTPγS binding	EC_{50} [nM]	870	1000 (G)	335	Guery,2007
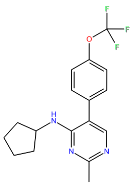	15	[^{35}S]-GTPγS binding	EC_{50} [nM]	4570	1000 (G)	337	Guery,2007
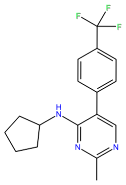	12	[^{35}S]-GTPγS binding	EC_{50} [nM]	5000	1000 (G)	321	Guery,2007

Structure	Name in litterature	Biolocical Data				MW g/mol	Source
		Experiment	Activity type	Activity value	[G] / [B] nM		
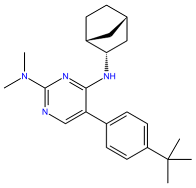	43	[^{35}S]-GTPγS binding	EC_{50} [nM]	1660	1000 (G)	365	Guery,2007
**Cluster3**							
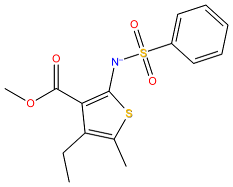	25	[^{35}S]-GTPγS binding	increase (%) relative to GABA, 25µM [cpd]	5.9	10000 (G)	339	Mugnaini,2013
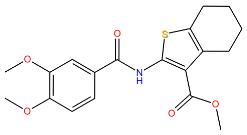	22	[^{35}S]-GTPγS binding	increase (%) relative to GABA, 25µM [cpd]	9	10000 (G)	375	Mugnaini,2013
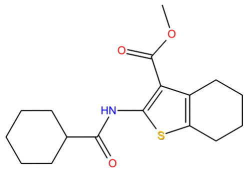	20	[^{35}S]-GTPγS binding	increase (%) relative to GABA, 25µM [cpd]	12	10000 (G)	321	Mugnaini,2013
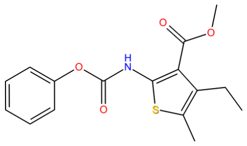	16	[^{35}S]-GTPγS binding	increase (%) relative to GABA, 25µM [cpd]	8.2	10000 (G)	319	Mugnaini,2013
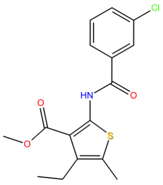	12	[^{35}S]-GTPγS binding	increase (%) relative to GABA, 25µM [cpd]	11	10000 (G)	338	Mugnaini,2013
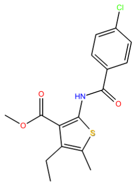	11	[^{35}S]-GTPγS binding	increase (%) relative to GABA, 2.5µM [cpd]	9.2	10000 (G)	338	Mugnaini,2013

Structure	Name in litterature	Biolocical Data				MW g/mol	Source
		Experiment	Activity type	Activity value	[G] / [B] nM		
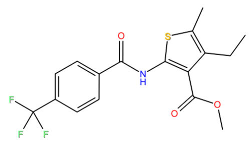	10	[^{35}S]-GTPγS binding	increase (%) relative to GABA, 2.5μM [cpd]	17.8	10000 (G)	371	Mugnaini,2013
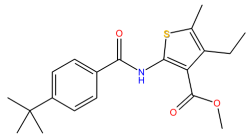	7	[^{35}S]-GTPγS binding	increase (%) relative to GABA, 2.5μM [cpd]	19	10000 (G)	359	Mugnaini,2013
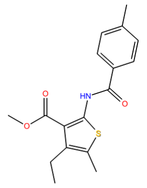	6	[^{35}S]-GTPγS binding	increase (%) relative to GABA, 25μM [cpd]	18.2	10000 (G)	317	Mugnaini,2013
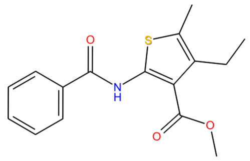	5	[^{35}S]-GTPγS binding	increase (%) relative to GABA, 25μM [cpd]	12	10000 (G)	303	Mugnaini,2013
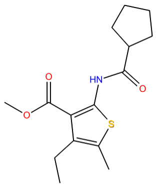	4	[^{35}S]-GTPγS binding	increase (%) relative to GABA, 25μM [cpd]	12.1	10000 (G)	295	Mugnaini,2013
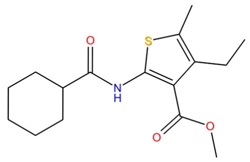	COR628	[^{35}S]-GTPγS binding	EC_{50} [nM]	970	10000 (G)	309	Castelli,2012
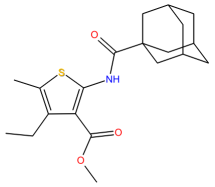	COR627	[^{35}S]-GTPγS binding	EC_{50} [nM]	910	10000 (G)	361	Castelli,2012

Structure	Name in litterature	Biolocical Data				MW g/mol	Source
		Experiment	Activity type	Activity value	[G] / [B] nM		
Cluster4							
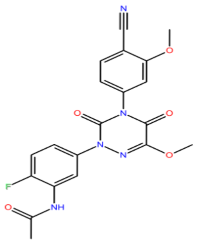	ADX71943	[35S]-GTPγS binding	EC_{50} [nM]	28	EC_{50} (B)	425	Kalinichev,2014
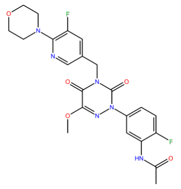	4.268	[35S]-GTPγS binding	EC_{50} [nM]	<100	EC_{50} (G or B)	488	Patent EP2662366A1
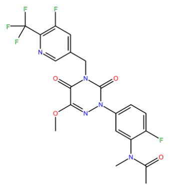	4.266	[35S]-GTPγS binding	EC_{50} [nM]	<100	EC_{50} (G or B)	485	Patent EP2662366A1
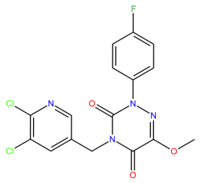	4.262	[35S]-GTPγS binding	EC_{50} [nM]	[100-500]	EC_{50} (G or B)	397	Patent EP2662366A1
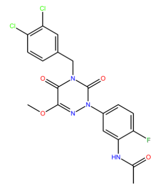	4.233	[35S]-GTPγS binding	EC_{50} [nM]	<100	EC_{50} (G or B)	453	Patent EP2662366A1
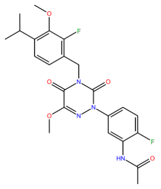	4.255	[35S]-GTPγS binding	EC_{50} [nM]	<100	EC_{50} (G or B)	474	Patent EP2662366A1
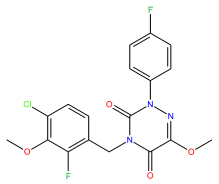	4.253	[35S]-GTPγS binding	EC_{50} [nM]	<100	EC_{50} (G or B)	410	Patent EP2662366A1

Structure	Name in litterature	Biolocical Data				MW g/mol	Source
		Experiment	Activity type	Activity value	[G] / [B] nM		
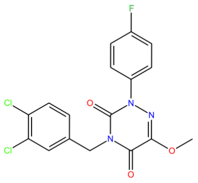	4.251	[^{35}S]-GTPγS binding	EC$_{50}$ [nM]	<100	EC$_{50}$ (G or B)	396	Patent EP2662366A1
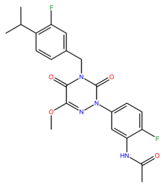	4.248	[^{35}S]-GTPγS binding	EC$_{50}$ [nM]	<100	EC$_{50}$ (G or B)	444	Patent EP2662366A1
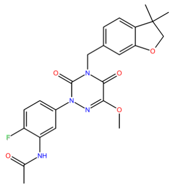	4.247	[^{35}S]-GTPγS binding	EC$_{50}$ [nM]	<100	EC$_{50}$ (G or B)	454	Patent EP2662366A1
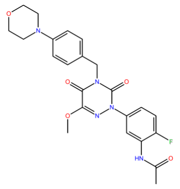	4.244	[^{35}S]-GTPγS binding	EC$_{50}$ [nM]	<100	EC$_{50}$ (G or B)	469	Patent EP2662366A1
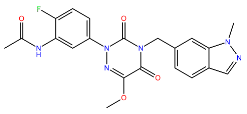	4.242	[^{35}S]-GTPγS binding	EC$_{50}$ [nM]	<100	EC$_{50}$ (G or B)	438	Patent EP2662366A1
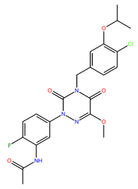	4.241	[^{35}S]-GTPγS binding	EC$_{50}$ [nM]	<100	EC$_{50}$ (G or B)	477	Patent EP2662366A1
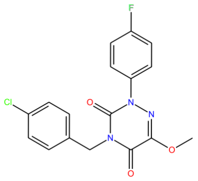	4.239	[^{35}S]-GTPγS binding	EC$_{50}$ [nM]	[500-1000]	EC$_{50}$ (G or B)	362	Patent EP2662366A1

Structure	Name in litterature	Biolocical Data				MW g/mol	Source
		Experiment	Activity type	Activity value	[G] / [B] nM		
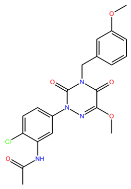	4.236	[35S]-GTPγS binding	EC_{50} [nM]	<100	EC_{50} (G or B)	431	Patent EP2662366A1
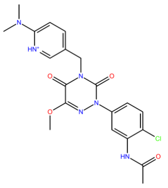	4.234	[35S]-GTPγS binding	EC_{50} [nM]	<100	EC_{50} (G or B)	445	Patent EP2662366A1
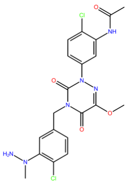	4.232	[35S]-GTPγS binding	EC_{50} [nM]	<100	EC_{50} (G or B)	479	Patent EP2662366A1
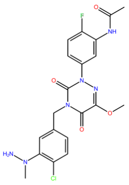	4.231	[35S]-GTPγS binding	EC_{50} [nM]	<100	EC_{50} (G or B)	463	Patent EP2662366A1
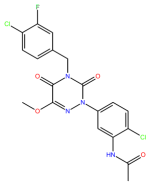	4.211	[35S]-GTPγS binding	EC_{50} [nM]	<100	EC_{50} (G or B)	453	Patent EP2662366A1
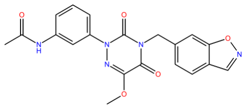	4.209	[35S]-GTPγS binding	EC_{50} [nM]	<100	EC_{50} (G or B)	407	Patent EP2662366A1
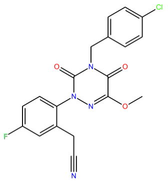	4.207	[35S]-GTPγS binding	EC_{50} [nM]	<100	EC_{50} (G or B)	401	Patent EP2662366A1

Structure	Name in litterature	Biolocical Data				MW g/mol	Source
		Experiment	Activity type	Activity value	[G] / [B] nM		
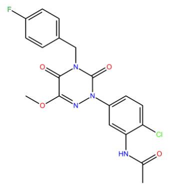	4.205	[^{35}S]-GTPγS binding	EC_{50} [nM]	[100-500]	EC_{50} (G or B)	419	Patent EP2662366A1
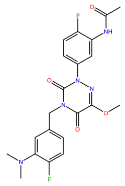	4.204	[^{35}S]-GTPγS binding	EC_{50} [nM]	<100	EC_{50} (G or B)	445	Patent EP2662366A1
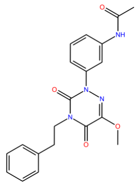	4.198	[^{35}S]-GTPγS binding	EC_{50} [nM]	[100-500]	EC_{50} (G or B)	380	Patent EP2662366A1
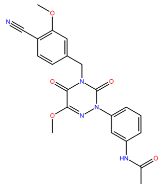	4.197	[^{35}S]-GTPγS binding	EC_{50} [nM]	<100	EC_{50} (G or B)	421	Patent EP2662366A1
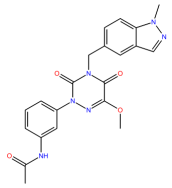	4.192	[^{35}S]-GTPγS binding	EC_{50} [nM]	[100-500]	EC_{50} (G or B)	420	Patent EP2662366A1
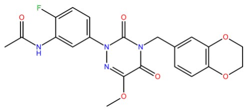	4.191	[^{35}S]-GTPγS binding	EC_{50} [nM]	[500-1000	EC_{50} (G or B)	442	Patent EP2662366A1
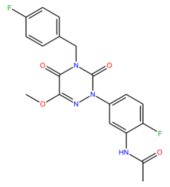	4.185	[^{35}S]-GTPγS binding	EC_{50} [nM]	[100-500]	EC_{50} (G or B)	402	Patent EP2662366A1

Structure	Name in litterature	Biolocical Data				MW g/mol	Source
		Experiment	Activity type	Activity value	[G] / [B] nM		
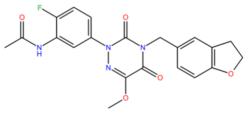	4.135	[^{35}S]-GTPγS binding	EC_{50} [nM]	[100-500]	EC_{50} (G or B)	426	Patent EP2662366A1
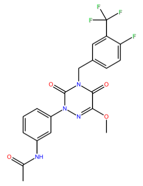	4.118	[^{35}S]-GTPγS binding	EC_{50} [nM]	<100	EC_{50} (G or B)	452	Patent EP2662366A1
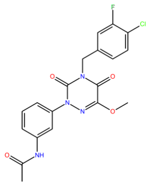	4.05	[^{35}S]-GTPγS binding	EC_{50} [nM]	<100	EC_{50} (G or B)	419	Patent EP2662366A1
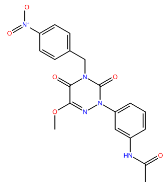	4.02	[^{35}S]-GTPγS binding	EC_{50} [nM]	[100-500]	EC_{50} (G or B)	411	Patent EP2662366A1
**Cluster5**							
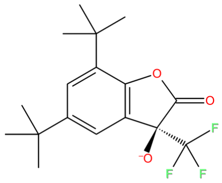	(+)-BHFF	Ca^{2+} intra mobilization	EC_{50} [nM]	80.6	10 (G)	330	Malherbe, 2008
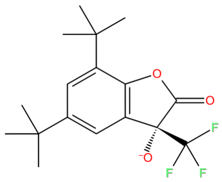	(-)-BHFF	Ca^{2+} intra mobilization	EC_{50} [nM]	177	10 (G)	330	Malherbe, 2008
	rac-BHFF	Ca^{2+} intra mobilization	EC_{50} [nM]	234	10 (G)	330	Malherbe, 2008

Structure	Name in litterature	Biolocical Data				MW g/mol	Source
		Experiment	Activity type	Activity value	[G] / [B] nM		
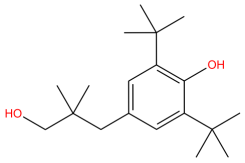	CGP7930	$[^{35}S]$-GTPγS binding	EC50 [nM]	4600	1000 (G)	292	Urwyler,2001
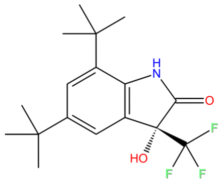	BHF1	Ca^{2+} intra mobilization	EC50 [nM]	289	10 (G)	345	Malherbe, 2008
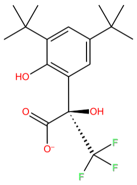	BHFHP	Ca^{2+} intra mobilization	EC50 [nM]	334	10 (G)	347	Malherbe, 2008
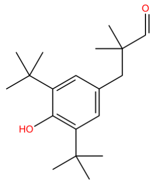	CGP13501	$[^{35}S]$-GTPγS binding	?	?	?	290	Urwyler, 2001

Castelli, M.P., Casu, A., Casti, P., Lobina, C., Carai, M.A.M., Colombo, G., Solinas, M., Giunta, D., Mugnaini, C., Pasquini, S., et al. (2012). Characterization of COR627 and COR628, Two Novel Positive Allosteric Modulators of the GABAB Receptor. J. Pharmacol. Exp. Ther. *340*, 529–538.
Guery, S., Floersheim, P., Kaupmann, K., and Froestl, W. (2007). Syntheses and optimization of new GS39783 analogues as positive allosteric modulators of GABAB receptors. Bioorg. Med. Chem. Lett. *17*, 6206–6211.

Kalinichev, M., Donovan-Rodriguez, T., Girard, F., Riguet, E., Rouillier, M., Bournique, B., Haddouk, H., Mutel, V., and Poli, S. (2014). Evaluation of peripheral versus central effects of GABAB receptor activation using a novel, positive allosteric modulator of the GABAB receptor ADX71943, a pharmacological tool compound with a fully peripheral activity profile. Br. J. Pharmacol. *171*, 4941–4954.

Malherbe, P., Masciadri, R., Norcross, R.D., Knoflach, F., Kratzeisen, C., Zenner, M.-T., Kolb, Y., Marcuz, A., Huwyler, J., Nakagawa, T., et al. (2008). Characterization of (R,S)-5,7-di-tert-butyl-3-hydroxy-3-trifluoromethyl-3H-benzofuran-2-one as a positive allosteric modulator of GABAB receptors. Br. J. Pharmacol. *154*, 797–811.

Mugnaini, C., Pedani, V., Casu, A., Lobina, C., Casti, A., Maccioni, P., Porcu, A., Giunta, D., Lamponi, S., Solinas, M., et al. (2013). Synthesis and Pharmacological Characterization of 2-(Acylamino)thiophene Derivatives as Metabolically Stable, Orally Effective, Positive Allosteric Modulators of the GABAB Receptor. J. Med. Chem. *56*, 3620–3635.

Urwyler, S., Mosbacher, J., Lingenhoehl, K., Heid, J., Hofstetter, K., Froestl, W., Bettler, B., and Kaupmann, K. (2001). Positive allosteric modulation of native and recombinant gamma-aminobutyric acid(B) receptors by 2,6-Di-tert-butyl-4-(3-hydroxy-2,2-dimethyl-propyl)-phenol (CGP7930) and its aldehyde analog CGP13501. Mol. Pharmacol. *60*, 963–971.

Urwyler, S., Pozza, M.F., Lingenhoehl, K., Mosbacher, J., Lampert, C., Froestl, W., Koller, M., and Kaupmann, K. (2003). N,N′-Dicyclopentyl-2-methylsulfanyl-5-nitro-pyrimidine-4,6-diamine (GS39783) and Structurally Related Compounds: Novel Allosteric Enhancers of γ-Aminobutyric AcidB Receptor Function. J. Pharmacol. Exp. Ther. *307*, 322–330.
