# Supplementary material for: Ligand-guided homology modelling of the GABAB2 subunit of the GABAB receptor
Source: PLoS One. 2017 Mar 21;12(3):e0173889. doi: 10.1371/journal.pone.0173889 (PMC5360267; doi:10.1371/journal.pone.0173889)
Supplement: S2 Table — (PDF) [file pone.0173889.s013.pdf]

S2 Table. Structures of experimentally known inactive ligands used as decoys.

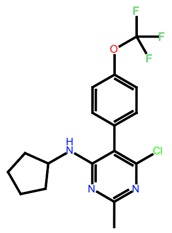

Inactive compound: 15a
Source: Guery 2007

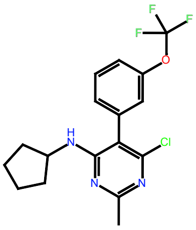

Inactive compound: 14a
Source: Guery 2007

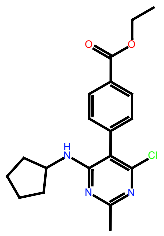

Inactive compound: 13a
Source: Guery 2007

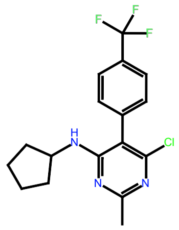

Inactive compound: 12a
Source: Guery 2007

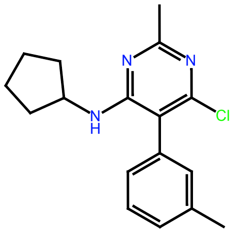

Inactive compound: 11a
Source: Guery 2007

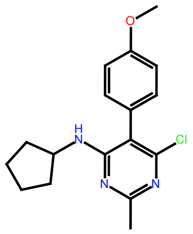

Inactive compound: 10a
Source: Guery 2007

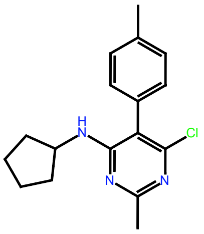

Inactive compound: 9a
Source: Guery 2007

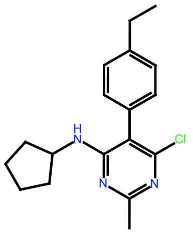

Inactive compound: 8a
Source: Guery 2007

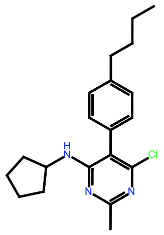

Inactive compound: 7a
Source: Guery 2007

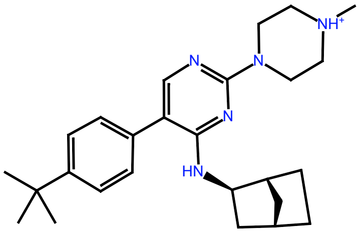

Inactive compound: 44
Source: Guery 2007

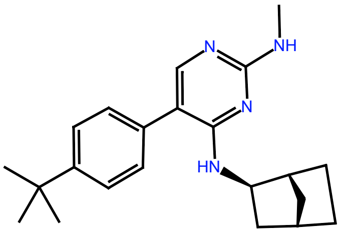

Inactive compound: 42
Source: Guery 2007

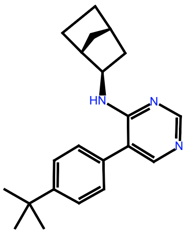

Inactive compound: 39
Source: Guery 2007

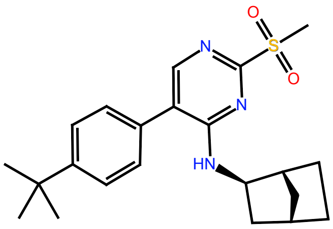

Inactive compound: 38
Source: Guery 2007

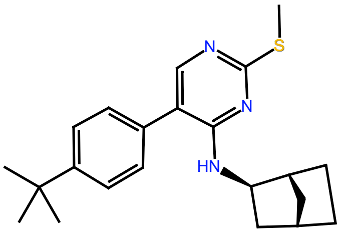

Inactive compound: 37
Source: Guery 2007

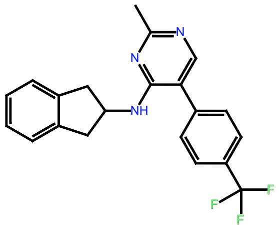

Inactive compound: 34
Source: Guery 2007

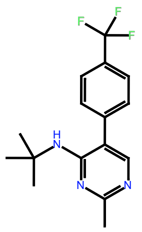

Inactive compound: 33
Source: Guery 2007

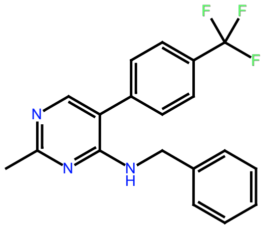

Inactive compound: 32
Source: Guery 2007

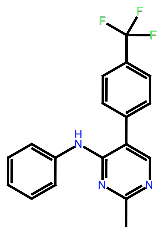

Inactive compound: 31
Source: Guery 2007

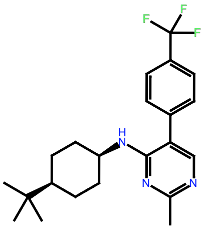

Inactive compound: 30
Source: Guery 2007

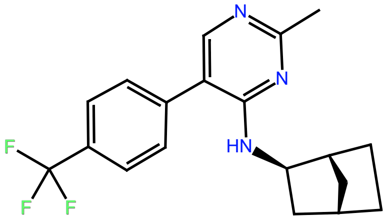

Inactive compound: 28
Source: Guery 2007

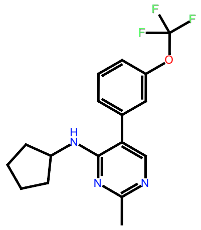

Inactive compound: 14b
Source: Guery 2007

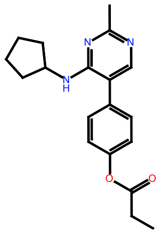

Inactive compound: 13b
Source: Guery 2007

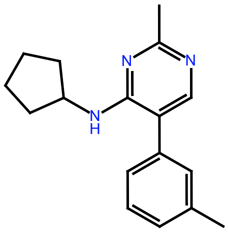

Inactive compound: 11b
Source: Guery 2007

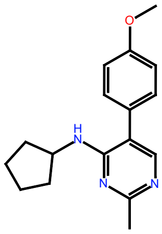

Inactive compound: 10b
Source: Guery 2007

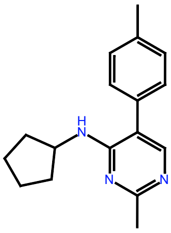

Inactive compound: 9b
Source: Guery 2007

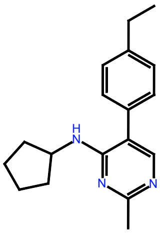

Inactive compound: 8b
Source: Guery 2007

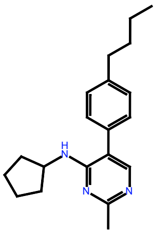

Inactive compound: 7b
Source: Guery 2007

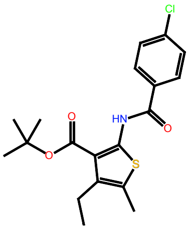

Inactive compound: 35
Source: Mugnaini 2013

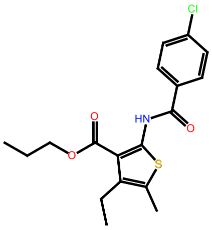

Inactive compound: 34
Source: Mugnaini 2013

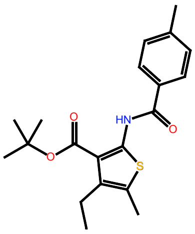

Inactive compound: 33
Source: Mugnaini 2013

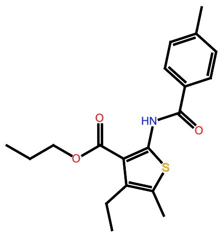

Inactive compound: 32
Source: Mugnaini 2013

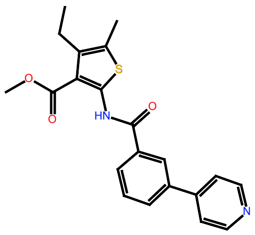

Inactive compound: 31
Source: Mugnaini 2013

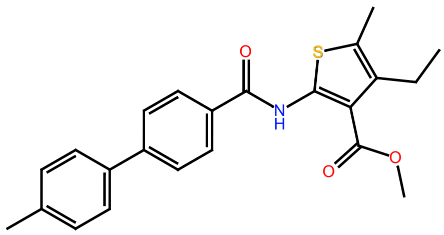

Inactive compound: 30
Source: Mugnaini 2013

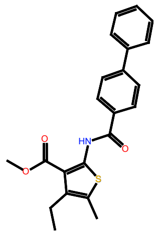

Inactive compound: 29
Source: Mugnaini 2013

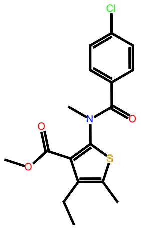

Inactive compound: 28
Source: Mugnaini 2013

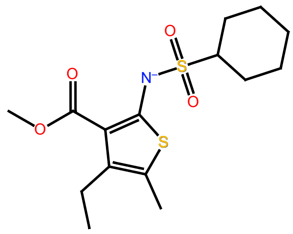

Inactive compound: 27
Source: Mugnaini 2013

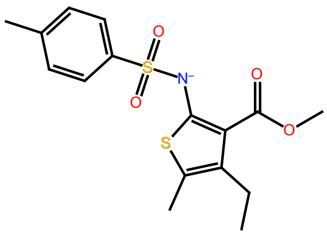

Inactive compound: 26
Source: Mugnaini 2013

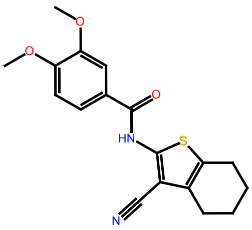

Inactive compound: 24
Source: Mugnaini 2013

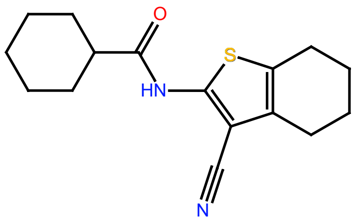

Inactive compound: 23
Source: Mugnaini 2013

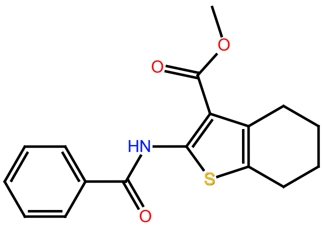

Inactive compound: 21
Source: Mugnaini 2013

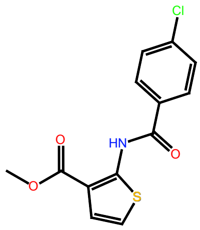

Inactive compound: 19
Source: Mugnaini 2013

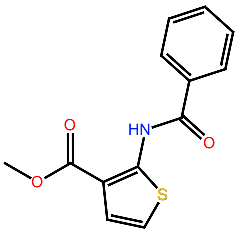

Inactive compound: 18
Source: Mugnaini 2013

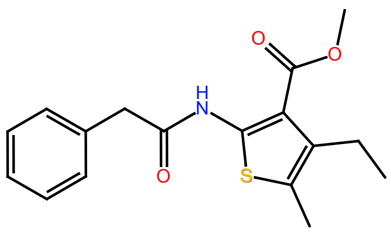

Inactive compound: 17
Source: Mugnaini 2013

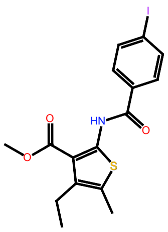

Inactive compound: 15
Source: Mugnaini 2013

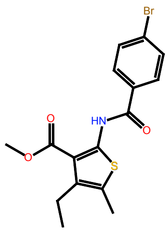

Inactive compound: 14
Source: Mugnaini 2013

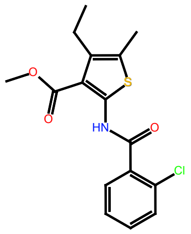

Inactive compound: 13
Source: Mugnaini 2013

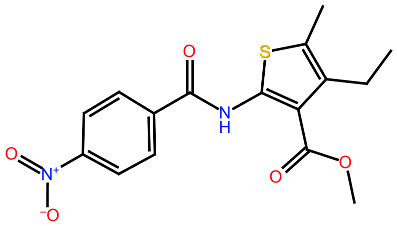

Inactive compound: 9
Source: Mugnaini 2013

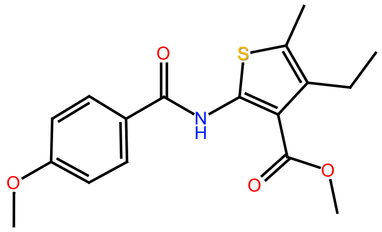

Inactive compound: 8
Source: Mugnaini 2013

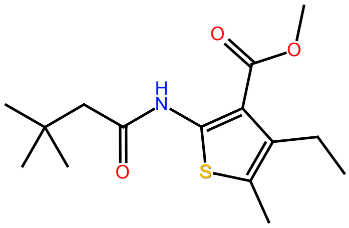

Inactive compound: 3
Source: Mugnaini 2013

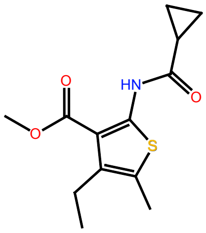

Inactive compound: 2
Source: Mugnaini 2013

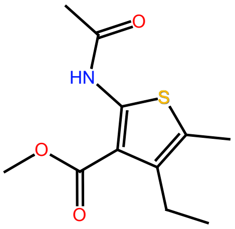

Inactive compound: 1
Source: Mugnaini 2013

Guery, S., Floersheim, P., Kaupmann, K., and Froestl, W. (2007). Syntheses and optimization of new GS39783 analogues as positive allosteric modulators of GABAB receptors. Bioorg. Med. Chem. Lett. *17*, 6206–6211.

Mugnaini, C., Pedani, V., Casu, A., Lobina, C., Casti, A., Maccioni, P., Porcu, A., Giunta, D., Lamponi, S., Solinas, M., et al. (2013). Synthesis and Pharmacological Characterization of 2-(Acylamino)thiophene Derivatives as Metabolically Stable, Orally Effective, Positive Allosteric Modulators of the GABAB Receptor. J. Med. Chem. *56*, 3620–3635.
