# Supplementary material for: Ligand-guided homology modelling of the GABAB2 subunit of the GABAB receptor
Source: PLoS One. 2017 Mar 21;12(3):e0173889. doi: 10.1371/journal.pone.0173889 (PMC5360267; doi:10.1371/journal.pone.0173889)
Supplement: S3 Table — See S1 Table for ligand activity data and structures. (PDF) [file pone.0173889.s014.pdf]

S3 Table. **Induced-fit docking (IFD) input ligands.** See S1 Table for ligand activity data and structures.

Cluster	Template				
	**1u19**	**2rh1**	**4k5y**	**4oo9**	**4or2**
**1**	CGA38493	GS39783	GS39783	CGA19414	CGP10773
**2**	43	43	43	29	29
**3**	10	20	7	25	25
**4**	4.185	4.118	4.255	4.135	4.244
**5**	BHFHP	BHFHP	CGP7930	BHF1	BHF1
