# Supplementary material for: Ligand-guided homology modelling of the GABAB2 subunit of the GABAB receptor
Source: PLoS One. 2017 Mar 21;12(3):e0173889. doi: 10.1371/journal.pone.0173889 (PMC5360267; doi:10.1371/journal.pone.0173889)
Supplement: S4 Table — 8 final models highlighted in red. 1U19, rhodopsin-based models; 4OR2, mGlu1-based models; 4OO9, mGlu5-based models; 4K5Y, CRF1R-based models; 2RH1, β2-AR-based models. (PDF) [file pone.0173889.s015.pdf]

S4 Table. BEDROC values of 10 best models per ligand cluster. 8 final models highlighted in red. 1U19, rhodopsin-based models; 4OR2, mGlu1-based models; 4OO9, mGlu5-based models; 4K5Y, CRF1R-based models; 2RH1, β_2-AR-based models.

Cluster1		Cluster2		Cluster3		Cluster4		Cluster5	
**Model name**	**BEDROC (α =20)**	**Model name**	**BEDROC (α =20)**	**Model name**	**BEDROC (α =20)**	**Model name**	**BEDROC (α =20)**	**Model name**	**BEDROC (α =20)**
**c1_m1_1u19**	**0.792**	c2_m3_4oo9	0.685	c3_m1_4k5y	0.395	c4_m3_4or2	0.894	**c5_m1_4oo9**	**0.999**
c1_m3_1u19	0.733	**c2_m1_4oo9**	**0.673**	c3_m2_4k5y	0.314	**c4_m1_4or2**	**0.851**	**c5_m2_4oo9**	**0.990**
**c1_m2_4oo9**	**0.616**	c2_m4_4or2	0.606	c3_m3_4or2	0.309	c4_m4_4oo9	0.847	c5_m3_4oo9	0.989
c1_m4_4oo9	0.483	c2_m5_4oo9	0.594	c3_m4_4or2	0.278	**c4_m2_4or2**	**0.841**	c5_m4_4oo9	0.978
c1_m5_1u19	0.460	**c2_m2_4oo9**	**0.575**	c3_m5_4oo9	0.274	c4_m5_1u19	0.83	c5_m5_4oo9	0.977
c1_m6_4or2	0.401	c2_m6_1u19	0.504	c3_m6_4oo9	0.263	c4_m6_4or2	0.811	c5_m6_4oo9	0.977
c1_m7_4oo9	0.401	c2_m7_4oo9	0.476	c3_m7_4or2	0.259	c4_m7_4or2	0.796	c5_m7_2rh1	0.972
c1_m8_1u19	0.370	c2_m8_4oo9	0.474	c3_m8_4oo9	0.254	c4_m8_1u19	0.795	c5_m8_4oo9	0.971
c1_m9_4oo9	0.301	c2_m9_4oo9	0.467	c3_m9_4or2	0.235	c4_m9_4or2	0.747	c5_m9_4oo9	0.970
c1_m10_4oo9	0.288	c2_m10_4k5y	0.457	c3_m10_4oo9	0.232	c4_m10_4or2	0.721	c5_m10_2rh1	0.963
