# Supplementary material for: Ligand-guided homology modelling of the GABAB2 subunit of the GABAB receptor
Source: PLoS One. 2017 Mar 21;12(3):e0173889. doi: 10.1371/journal.pone.0173889 (PMC5360267; doi:10.1371/journal.pone.0173889)
Supplement: S5 Table — (PDF) [file pone.0173889.s016.pdf]

S5 Table. BEDROC values and Glide score ranges from docking of cluster 1-5 PAMs into the 8 final $GABA_{B2}$ homology models.

Model name	Cluster1			Cluster2			Cluster3			Cluster4			Cluster5		
	BEDROC	docked # /11	Score range	BEDROC	docked # /10	Score range	BEDROC	docked # /13	Score range	BEDROC	docked # /33	Score range	BEDROC	docked # /6	Score range
c1_m1_ 1u19	0.792	11	-7.69 to -9.49	0.015	8	-4.36 to -6.80	0.139	12	-2.69 to -7.98	0.000	18	-2.98 to -5.86	0.003	1	-6.19 to -6.19
c1_m2_ 4oo9	0.616	10	-7.52 to -9.28	0.258	6	-6.87 to -9.05	0.036	8	-4.25 to -7.58	0.000	1	-5.52 to -5.52	0.029	2	-6.86 to -7.49
c2_m1_ 4oo9	0.000	11	-6.30 to -7.17	0.673	9	-8.17 to -10.07	0.047	13	-5.03 to -8.62	0.152	19	-5.81 to -9.23	0.022	6	-6.67 to -7.97
c2_m2_ 4oo9	0.007	11	-5.55 to -7.83	0.575	9	-7.86 to -10.23	0.010	10	-4.88 to -8.12	-	-	-	0.117	5	-7.96 to -8.63
c4_m1_ 4or2	0.000	11	-5.66 to -7.18	0.001	6	-5.85 to -7.76	0.053	13	-6.51 to -8.73	0.851	32	-8.24 to -11.00	0.000	5	-6.44 to -7.53
c4_m2_ 4or2	0.000	11	-5.84 to -6.63	0.000	9	-5.26 to -6.95	0.001	13	-5.73 to -8.10	0.841	32	-8.10 to -13.15	0.000	6	-5.99 to -6.68
c5_m1_ 4oo9	0.078	11	-4.06 to -7.10	0.234	4	-6.67 to -8.41	0.026	9	-3.51 to -7.15	-	-	-	0.999	6	-9.13 to -10.19
c5_m2_ 4oo9	0.288	11	-5.72 to -8.49	0.152	5	-6.59 to -8.10	0.075	8	-5.33 to -8.01	-	-	-	0.990	6	-9.03 to -9.94
