# Supplementary material for: Ligand-guided homology modelling of the GABAB2 subunit of the GABAB receptor
Source: PLoS One. 2017 Mar 21;12(3):e0173889. doi: 10.1371/journal.pone.0173889 (PMC5360267; doi:10.1371/journal.pone.0173889)
Supplement: S6 Table — Amino acids within 4 Å of more than 50% of the ligands are listed. SIFt(8), SIFt(7) general SIFt profiles; SIFt(C1-C5), cluster-specific SIFt profiles; SIFt(1U19-4OR2-4OO9), template-specific SIFt profiles. GPCRdb (C), class C GPCR database numbering scheme positions. (PDF) [file pone.0173889.s017.pdf]

S6 Table. SIFt profiles. Amino acids within 4 Å of more than 50 % of the ligands are listed. SIFt(8), SIFt(7) general SIFt profiles; SIFt(C1-C5), cluster-specific SIFt profiles; SIFt(1U19-4OR2-4OO9), template-specific SIFt profiles. GPCRdb (C), class C GPCR database numbering scheme positions.

GPCRdb (C)	$GABA_{B2}$	SIFt (8)	SIFt (7)	SIFt (C1)	SIFt (C2)	SIFt (C5)	SIFt (1U19)	SIFt (4OR2)	SIFt (4OO9)
3.32x32	R556							0.73	
3.33x33	T557	0.67	0.74		1			0.92	
3.36x36	L560	1	1	1	1	1	1	1	1
3.37x37	T561	0.7	0.67	0.76	1	1	1	0.55	0.88
3.40x40	Y564	1	1	1	1	1	1	1	1
3.41x41	T565	0.62	0.58	0.52		0.58	1	0.83	
3.44x44	F568	0.71	0.79		0.5			0.98	
4.46x46	I611			0.52			1		
4.47x47	L612			0.52			1		
4.50x50	W615						0.91		
ECL2	H647						0.82		
ECL2	E649			0.52			1		
ECL2	N650			0.52			1		
5.40x40	W656	0.6	0.56	0.52	0.94	0.5	1	0.55	0.57
5.41x41	L657	0.9	1		1	1		1	1
5.42x42	I659			0.52			1		
5.43x43	V660	0.63	0.68		1	1		0.56	0.88
5.44x44	Y661	0.9	1		1	1		1	1
5.46x46	Y663			0.52			1		
5.47x47	K664	0.99	1	0.95	1	1	0.91	1	1
5.48x48	G665	0.57	0.63					0.98	
5.50x50	L667		0.52					0.84	
5.51x51	M668	0.75	0.83		1			1	0.55
6.46x46	V699	0.9	1		1	1		1	1
6.49x49	M702	1	1	1	1	1	1	1	1
6.5x50	C703	1	1	1	1	1	1	1	1
6.53x53	G706	0.97	0.96	1	1	1	1	0.94	1
6.54x54	A707	0.9	0.88	1		1	1	1	0.7
6.56x56	V709			0.57	0.5		1		
6.57x57	S710	1	1	1	1	1	1	1	1
7.28x29	Q720				0.72				
7.29x30	F721			0.52			1		
7.31x32	I723	0.68	0.75		1	1		0.61	0.97
7.32x33	V724	1	1	1	1	1	1	1	1
7.33x34	A725			0.52			1		
7.35x36	V727	0.9	1		1	1		1	1
7.36x37	I728	0.69	0.65	1	0.56	1	1	0.56	0.8
7.39x40	C731	0.98	1	0.9	1	1	0.82	1	1
